# Mixed halide perovskites for spectrally stable and high-efficiency blue light-emitting diodes

**DOI:** 10.1038/s41467-020-20582-6

**Published:** 2021-01-13

**Authors:** Max Karlsson, Ziyue Yi, Sebastian Reichert, Xiyu Luo, Weihua Lin, Zeyu Zhang, Chunxiong Bao, Rui Zhang, Sai Bai, Guanhaojie Zheng, Pengpeng Teng, Lian Duan, Yue Lu, Kaibo Zheng, Tönu Pullerits, Carsten Deibel, Weidong Xu, Richard Friend, Feng Gao

**Affiliations:** 1 Department of Physics, Chemistry and Biology (IFM), Linköping University, Linköping, Sweden; 2 Cavendish Laboratory, University of Cambridge, Cambridge, UK; 3 Institut für Physik, Technische Universität Chemnitz, Chemnitz, Germany; 4 Key Lab of Organic Optoelectronics and Molecular Engineering of Ministry of Education, Department of Chemistry, Tsinghua University Beijing, Beijing, China; 5 Chemical Physics and NanoLund, Lund University, Box 124, 22100 Lund, Sweden; 6 Institute of Microstructure and Property of Advanced Materials, Faculty of Materials and Manufacturing, Beijing University of Technology, Beijing, 100124 China; 7 Department of Chemistry, Technical University of Denmark, DK-2800 Kongens Lyngby, Denmark

**Keywords:** Electronic devices, Lasers, LEDs and light sources

## Abstract

Bright and efficient blue emission is key to further development of metal halide perovskite light-emitting diodes. Although modifying bromide/chloride composition is straightforward to achieve blue emission, practical implementation of this strategy has been challenging due to poor colour stability and severe photoluminescence quenching. Both detrimental effects become increasingly prominent in perovskites with the high chloride content needed to produce blue emission. Here, we solve these critical challenges in mixed halide perovskites and demonstrate spectrally stable blue perovskite light-emitting diodes over a wide range of emission wavelengths from 490 to 451 nanometres. The emission colour is directly tuned by modifying the halide composition. Particularly, our blue and deep-blue light-emitting diodes based on three-dimensional perovskites show high EQE values of 11.0% and 5.5% with emission peaks at 477 and 467 nm, respectively. These achievements are enabled by a vapour-assisted crystallization technique, which largely mitigates local compositional heterogeneity and ion migration.

## Abstract (web summary)

Achieving bright and efficient blue emission in metal halide perovskite light-emitting diodes has proven to be challenging. Here, the authors demonstrate high EQE and spectrally stable blue light-emitting diodes based on mixed halide perovskites, with emission from 490 to 451 nm by using a vapour-assisted crystallization technique.

## Introduction

Blue light-emitting diodes, with the Commission Internationale de l’Eclairage (CIE) y coordinate value below 0.15 along with the (*x* + *y*) value below 0.30, are of critical importance for display and energy-saving lighting applications^1^. Similar to preceding light-emitting technologies, achieving efficient blue emission in metal halide perovskite light-emitting diodes (PeLEDs) has proven to be very challenging, with performance lagging far behind their green, red and near-infrared counterparts^2–7^. Current efforts in blue PeLEDs largely take advantage of quantum confinement effects for bandgap engineering, i.e. using mixed dimensional perovskites or colloidal perovskite nanocrystals^8–10^. Although impressive progress has been achieved in developing sky-blue PeLEDs (with CIEy > 0.15) (Supplementary Table 1)^11^, there are increasing difficulties to realize blue emission using these strategies. For example, state-of-the-art blue perovskite emitters achieved by strong quantum confinement commonly suffer from deteriorated electronic properties due to an excess of large-size organic cations and/or over-capped ligands. These issues lead to problematic charge injection and hence low brightness, as well as a big gap between photoluminescence quantum yields (PLQYs) of thin films and external quantum efficiencies (EQEs) of devices^12–14^.

Compared with enhancing quantum confinement, modulating the halide anions is a more straightforward way to tune the bandgap of perovskites^15^. However, implementation of this facile approach in blue PeLEDs is largely hindered by poor colour stability of the resultant blue perovskite emitters (mixed bromide/chloride perovskites), due to anion segregation under electric bias^16–18^. In addition, it has been widely observed that the PLQYs decrease with increasing chloride content since chloride perovskites are less defect-tolerant compared to their bromide and iodide counterparts^19,20^. Both issues are particularly pronounced in perovskites with the high chloride content that is desired for producing blue and deep-blue emssion^19–22^. Very recently, strategies on mitigating photo-induced phase segregation in perovskite solar cells (*e.g*. defect passivation) have been borrowed to improve the spectral stability of mixed bromide/chloride blue PeLEDs^23,24^. These strategies were so far demonstrated to be feasible only in the cases where the chloride content is low (<30%)^22,25^. Unfortunately, even by combining the strategies of mixed bromide/chloride perovskites with the advantages of enhanced quantum confinement, device performance of spectrally stable blue PeLEDs is still far from practical applications (Supplementary Table 2)^11,14,20,25^.

Here, we demonstrate that blue PeLEDs based on mixed halide perovskites can be highly efficient and their colour instability issues can be substantially eliminated across a large range of the blue spectral region spanning 490–451 nm (with a high chloride content ranging from 30% to 57%), without any assistance from enhanced quantum confinement. We show that not only halide ion migration, but also compositional heterogeneity, is critical for triggering phase segregation. Both factors can be remarkably suppressed through depositing the perovskite films via a vapour-assisted crystallization (VAC) technique. As a result, we demonstrate spectrally stable blue PeLEDs presenting a high EQE value of 11.0% and a peak brightness of 2180 cd m^−2^, with an emission peak at 477 nm and CIE coordinates of (0.107, 0.115). In addition, we fabricate a PeLED exhibiting ideal deep-blue emission at 467 nm and a decent EQE of 5.5%, which is the highest efficiency with an emission peak below 470 nm. The CIE coordinates of our deep-blue PeLEDs are (0.130, 0.059), approaching that of Rec. 2020 specified primary blue.

## Results and discussion

### Device fabrication and spectral stability

We prepare perovskites from precursors with a stoichiometry of Cs^+^: FA^+^: Pb^2+^: [Br_1-x_ + Cl_x_]^−^ = 1.2: 0.3: 1: 3.5 (x = 30%–57%), where FA^+^ is formamidinium. In the cases of x below 20%, we do not observe any colour instability issues (Supplementary Fig. 1). We focus our discussions on the perovskites from a precursor solution with x = 40%, which is the most representative case due to its high chloride content, decent device performance and emission within the blue region. The chloride content in this film accounts for 42% of total halide anions as determined by X-ray photoelectron spectroscopy (XPS) (Supplementary Fig. 2). We introduce 4,7,10-trioxa-1,13-tridecanediamin (TTDDA) into the perovskite precursors as a passivating agent to reduce defects^5^.

**Fig. 1:**
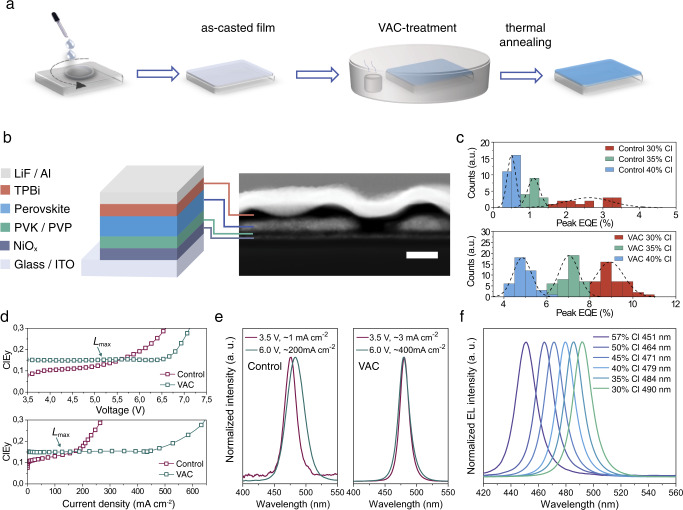
Device fabrication and characteristics. **a** An illustration of the VAC-treatment. **b** Schematic of the PeLED structure and the HAADF cross-sectional device image. The scale bar is 100 nm. **c** Histograms of peak EQEs extracted from control (top) and VAC-treated devices (bottom) with varying chloride contents (30%, 35% and 40%). **d**–**f** Spectral stability for control and VAC-treated devices with 40% Cl loading. The representative plots of CIE_y_ versus applied voltages (top) and current densities (bottom) (**d**); EL spectra at low and high voltage/current density for control (left) and VAC-treated devices (right) (**e**); EL spectra of VAC-treated devices with varying chloride content (30–57%) at maximum luminance (**f**). The points labelled as *L*_max_ in (**d**) represent the operational condition for peak luminance.

We show an illustration of the VAC-treatment for preparation of perovskite films in Fig. 1a. In brief, the as-casted films are directly moved into a petri-dish with a dimethylformamide (DMF) atmosphere, followed by a typical thermal annealing process. Control samples, annealed directly after spin-coating, are prepared for comparison. The different film processing techniques result in distinct variations of the film morphology, i.e. a discontinuous network of large grains for the VAC-treated films and full coverage of small nano-grains for the control ones, as shown in scanning electron microscope (SEM) images (Supplementary Fig. 3). We observe no change in the 3D crystal structure but a more preferential crystal orientation along the (110) direction and slight enhancement of crystallinity with VAC-treatment, as demonstrated by grazing-incidence wide-angle X-ray scattering (GIWAXS) and X-ray diffraction (XRD) measurements (Supplementary Fig. 4).

We fabricate the PeLEDs based on a device structure of indium tin oxide (ITO)/nickel oxide (NiO_x_, 10 nm)/ poly(9-vinylcarbazole) (PVK): polyvinylpyridine (PVP) (10 nm)/ perovskite/ 2,2′,2″-(1,3,5-benzinetriyl)tris(1-phenyl-1H-benzimidazole) (TPBi)/ lithium fluoride (1 nm)/aluminium (100 nm) (Fig. 1b). The employment of NiO_x_/PVK bilayer facilitates hole injection due to a cascade energy level alignment^26^, which contributes to improved PeLED performance (Supplementary Figs. 5 and 6). The PVP layer is used to improve the wettability of the precursor solution on the PVK surface, as demonstrated by the reduced water contact angle after PVP deposition (Supplementary Fig. 7). The high-angle annular dark-field scanning transmission electron microscope (HAADF-STEM) and energy-dispersive X-ray spectroscopy (EDX) device cross-sectional images indicate higher TPBi layer thickness (~50 nm) on the bottom hole injection layer with respect to that on perovskite grains (~35 nm) (Fig. 1b and Supplementary Fig. 8 (carbon distribution)), leading to enhanced local resistance at the TPBi/PVK:PVP interface. Combined with the large injection barrier caused by the energy level mismatch between TPBi and PVK, the discontinuous morphology in VAC-treated devices does not necessarily lead to strong electrical shunts under normal PeLED operational conditions^3,5^ (Supplementary Fig. 9). Notably, the VAC-treated devices with varying chloride content (30–40%) show considerable enhancement of EQE values compared to the respective control samples (Fig. 1c). We show the characteristics of the representative devices in Supplementary Fig. 10. We notice that, as expected, our device performance also benefits from the efficient defect passivation ability of TTDDA (Supplementary Fig. 11).

The VAC-treated devices show stable electroluminescence (EL) with a negligible shift of the CIE coordinates up to ~ 400 mA cm^−2^ (6–6.5 V), which is far above maximum light output (*L*_max_) conditions at 100 mA cm^−2^/5.0 V (Fig. 1d). The EL spectrum obtained at a high voltage (or current density) of 6 V (~400 mA cm^−2^) is only slightly broader than that at 3.5 V (~3 mA cm^−2^) (Fig. 1e). We attribute this slight EL broadening to charge carrier/phonon interaction due to Joule heating^27^, as similar behaviour is observed in pure-bromide PeLEDs (Supplementary Fig. 12). In contrast, the control devices undergo distinct emission colour changes starting at very low bias and current density of around 3.0–3.5 V and 1–5 mA cm^−2^ (Fig. 1d, e), analogous to previous reports on spectrally unstable mixed bromide/chloride PeLEDs^16,21^. Under harsh operational conditions, that is, with a bias larger than 6.5 V, distinct colour change is observable even in VAC-treated devices. We emphasize that in this case, the current density is over 400 mA cm^−2^, which is far above normal working conditions of reported blue PeLEDs^11,14,20,25^. Notably, we observe abnormal plateau-like *J–V* characteristics during the voltage sweep at 6–6.5 V and severe PL quenching after the operation (Supplementary Fig. 13), indicating severe device damage due to perovskite and/or interfacial degradation. Given the high current density, Joule heating could be a critical reason^28^.

With VAC-treatment, we demonstrate spectrally stable PeLEDs with emission colours from sky-blue to deep-blue (emission peaks at 490–451 nm) by simply varying the chloride content (30%–57%) (Fig. 1f and Supplementary Fig. 14). We also examine the spectral stability of our devices at a constant current density of 5 mA cm^−2^ (with initial luminance ranging from ~200 to ~600 cd m^−2^ for different devices). Although the operational lifetime is no better than those in previously reported blue PeLEDs (with T_50_ around 1–2 min)^8,11,12,20^, we observe no spectral shift even after 10 min of operation (Supplementary Fig. 15). Previous reports on photo-induced phase segregation in mixed halide perovskites indicate that it is only triggered when the excitation density is above a certain threshold, below which little to no effects are present^29,30^. Our results are consistent with these observations, indicating that employing mixed halide anions is a feasible approach for blue PeLEDs as long as we can control the phase segregation threshold to be far above working conditions.

### The origin of improved spectral stability

Although the underlying reason for phase segregation is complicated, previous investigations on perovskite solar cells propose that three factors may be collectively contributing to this phenomenon. These three factors include a polaron induced strain effect^31^, a thermodynamic process as driven by free energy differences associated with composition and band offsets^32^, and field-dependent anion motion^33^. We carry out a series of characterizations to understand the origin of the spectral stability of PeLEDs based on VAC-treated perovskite films.

**Fig. 2:**
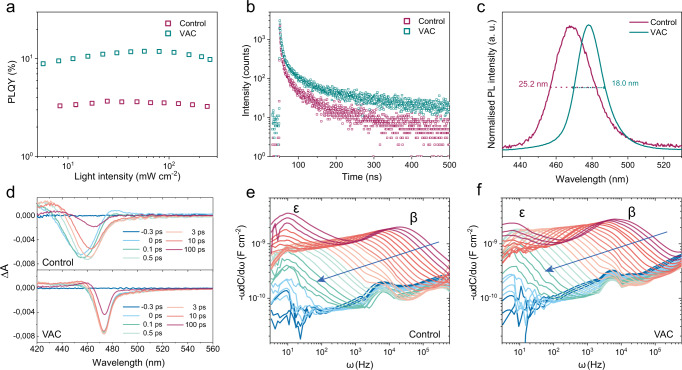
Understanding superior spectral stability of VAC-treated devices. **a**–**d** Photophysical characterizations for control and VAC-treated perovskite films: Fluence-dependent PLQYs (**a**); PL decay measured by TCSPC (**b**). PL spectra (**c)**; Transient absorption of control (top) and VAC-treated films (bottom) after excitation at 400 nm (**d**). **e**, **f** Derivatives of temperature-dependent capacitance versus frequency plots for control (**e**) and VAC-treated (**f**) devices. The blue arrows indicate temperature change from 350 K to 200 K. Here, two mobile ions marked as β and ε are visible.

We first measure PL properties of our perovskite films. We observe obviously enhanced PLQYs in the VAC-treated films across a wide range of excitation fluences, with a peak PLQY of 12% compared to 3% for the control sample (Fig. 2a). Time correlated single photon counting (TCSPC) measurements demonstrate a prolonged PL lifetime for VAC-treated samples compared to the control one (Fig. 2b). These results suggest fewer defects and much suppressed non-radiative recombination in the VAC-treated films, consistent with the higher EQEs of the devices. We believe that the enhanced spectral stability in our PeLEDs is partially ascribed to the reduced defects, as defects are generally believed to act as channels for anion hopping and hence facilitate phase segregation^34^.

In addition to the reduced defect density, we also observe significantly improved local compositional homogeneity in the VAC-treated films compared to the control sample. It is first evidenced by a steeper edge of the absorption spectrum (Supplementary Fig. 16a) and a much-narrowed PL linewidth (with full-width at half-maximum (FWHM) of ~18 nm) of VAC-treated films compared to that of control samples (FWHM of ~25 nm) (Fig. 2c). To gain further understanding of electronic states in the films, we conducted transient absorption (TA) spectroscopy measurements. The control film displays a broad photobleaching peak that shifts from 455 nm to 465 nm (Fig. 2d and Supplementary Figs. 16b, c), which is consistent with the coexistence of different phases. As there is no sign of low-dimensional phases from GIWAXS and XRD patterns, we assign the compositional heterogeneity in the control films to a non-uniform distribution of halide anions, which has been widely reported in bromide/iodide mixed perovskites^35,36^. In contrast, the VAC-treated film shows a single narrow ground state photobleaching situated at 473 nm, indicating a high compositional homogeneity. According to current polaronic and thermodynamic models for rationalizing phase segregation in perovskite solar cells, a high compositional heterogeneity can contribute to the phase segregation^31,32,37–39^. In specific, fluctuations in halide compositions can yield heterogeneous regions in the perovskites where polarons tend to localize at lower bandgap areas, leading to enhanced local lattice strain which drives de-mixing of halide anions^31,38,39^. A system with initially high free energy due to severe compositional disorder might be energetically unfavourable for phase stability as indicated by the thermodynamic model^37^. Lattice mismatch and discrepancy of band offsets between different phases are also believed to be the driving forces for phase segregation^32,37^. Our observations are in line with these previous investigations, demonstrating the critical role of high homogeneity in improving phase stability of VAC-treated devices.

Assured about the reduced defects and improved compositional homogeneity in VAC-treated films, we then evaluate field-dependent ion migration in our devices. We perform temperature-dependent admittance spectroscopy, from which we can determine ion migration activation energy (*E*_A_), ion diffusion coefficient (*D*), and concentration of mobile ions (*N*_i_)^40^. The capacitance (C) response of mobile ionic species can be probed by varying the frequency (ω) of an applied alternating voltage and the temperature. We show the admittance spectra in Supplementary Fig. 17 and the plots of derivatives (−ωdC/dω versus ω) in Fig. 2e, f. Two distinct signatures from mobile ionic species are visible, which are labelled ε and β. We confirm that the charge transport layers are not responsible for these signatures by characterizing devices with only charge transport layers (Supplementary Fig. 18)^41^. In particular, we observe that the response peaks at the low-frequency region (ε) in the VAC-treated devices are much less prominent than those in the control devices, suggesting a much lower mobile ion concentration. We show the deduced Arrhenius plots in Supplementary Fig. 19 and summarize all the obtained parameters (*E*_A_, *D*, and *N*_i_) in Supplementary Table 3. The *E*_A_ values of ion diffusion for both ε and β are very close in the two samples, implying that the mode of ion migration is not significantly altered. Both the concentration of mobile ions and ion diffusion coefficient are reduced in VAC-treated devices compared to the control devices. The most striking difference occurs to *N*_i_(ε), which is decreased from 5.4 × 10^16^ cm^−3^ to 1.6 × 10^16^ cm^−3^. Considering the small *E*_A_ of ε (~0.2 eV), we assign them to mobile halide anions^33,40^. The mitigated halide migration can be a result of reduced ionic defects, as confirmed by PLQYs and TCSPC results^34^.

Based on the analysis above, we conclude that the excellent spectral stability in VAC-treated devices originates from a synergistic effect of less ionic defects, mitigated ion migration and a higher compositional homogeneity.

### Understanding the effect of the VAC-treatment process

Having understood the origin of high colour stability and excellent device performance, the question that remains is how the VAC-treatment brings about these effects. We conduct SEM measurements to track the grain growth and morphological evolution of the films during the VAC treatment. We clearly observe two stages. The first stage happens within the first minute of vapour treatment, showing a crystal growth from initially formed small grains into large ones, accompanied by the morphological evolution from dense films into a discontinuous network (Supplementary Fig. 20). Considering the presence of crystalline perovskites within the pristine films and the diffusive vapour atmosphere, we assign the process of grain growth to Ostwald ripening. The wet films preserved by DMF vapour can be regarded as a sol system, with the solvent as the dispersing medium and perovskites as the dispersed phases. The ripening process occurs because large grains are more energetically favoured to smaller grains, leading to reduced grain boundaries and hence fewer defects. The second stage happens during the prolonged duration of treatment, which only has a slight impact on the morphology.

**Fig. 3:**
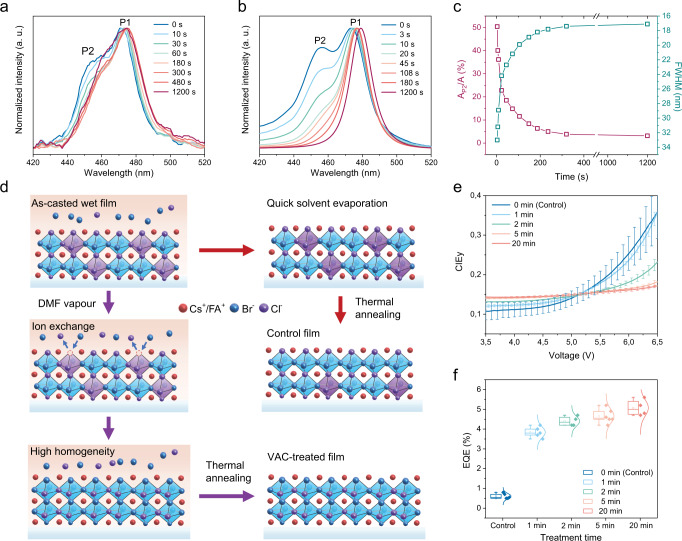
Understanding the halide redistribution during VAC-process. **a, b** PL evolution of the precursor films kept in the glovebox atmosphere (**a**) and DMF atmosphere (**b**) with time. **c** the evolution of emission linewidth and the proportion of P2 (A_P2_) to the respective total area of the emission band (A) in VAC-treated films with time. **d** Schematic illustration of the proposed mechanism for halide redistribution. Here, the purple Pb(Br/Cl)_6_^4−^ octahedra represent chloride-rich phases in respect to that with stoichiometric bromide/chloride distribution (blue octahedra). The khaki represents the liquid phase within the films and the blue arrows represent ion exchange process. The excessive ions within the dried films are not illustrated for clarity. **e**, **f** The evolution of CIE coordinates upon bias (**e**) and peak EQEs of the devices with varying duration of VAC-treatment (0, 1, 2, 5, 20 min). **f** The error bars present the standard deviation extracted from 4 to 6 devices.

We also employ in-situ PL and transmittance measurements to monitor the crystal growth with and without DMF vapour. The measurement setups are illustrated in Supplementary Fig. 21. Initially, both films show broad emission bands with the main peak at the low-energy region and a distinguishable shoulder at the high-energy region, which are labelled as P1 and P2, respectively (Fig. 3a, b). We speculate that the high-energy emission originates from initially formed Cl-rich perovskite phases due to their fast nucleation and crystallization, as governed by their poor solubility compared to Br-rich counterparts. By following the PL spectral evolution with time, we observe a gradual disappearance of P2 and a continuous red-shift of P1 in VAC-treated samples, leading to a narrow and single-emission peak eventually (Fig. 3b). To further clarify the spectral evolution of VAC-treated films in different time scales, we show the changes of emission bandwidth as well as the proportion of P2 (A_P2_) to a total area of emission band (A) with time in Fig. 3c. We find that the most striking changes occur within the first five minutes of treatment, the while prolonged duration of up to 20 min results in only a small difference (Fig. 3c). This PL evolution is consistent with the results of in-situ transmittance measurements, i.e. a red-shift of absorption onset and steeper absorption edge after VAC-treatment (Supplementary Fig. 22a). In contrast, keeping the pristine films in the glovebox atmosphere does not change the PL (Fig. 3a) and transmittance (Supplementary Fig. 22b) spectra to any significant degree over time.

Based on the in-situ spectroscopic measurement results, we can now rationalize the effect of the VAC-treatment. It provides a favourable diffusive environment for halide rearrangement within the films (Fig. 3d), which undergo an equilibrating crystallization process that homogenises local chemical composition and reduces disorder. Initially, the as-casted films are composed of various Cl-rich solid phases and Br-rich components in liquid phases due to nonequilibrium grain growth during spin-casting. For the films with no vapour atmosphere, quick solvent evaporation and following fast crystallization result in immediate freezing of the perovskite composition. The post-annealing could mitigate phase heterogeneity to some extent, as indicated by the weakened emission shoulder at short wavelength (P2) after annealing (Fig. 2c). However, the initially formed heterogeneous phases are still partially preserved in the resulting films. In contrast, with the presence of DMF vapour, the liquid phase can be preserved for a long duration. This facilitates and prolongs the following halide exchange process as driven and modulated by the chemical potential difference between solid (Cl-rich) and liquid phases (Br-rich), resulting in a rearranged composition that gradually approaches chemical equilibrium and homogeneous distribution of constituents. The following annealing procedure has little impact on the PL spectra of VAC-treated films, further confirming that the high homogeneous composition has already been achieved during the VAC-treatment.

We then tune the duration of the DMF vapour treatment to assess the impact on spectral stability and device efficiency in different timescale (Fig. 3e, f), further supporting our understanding of this technique. We observe distinct batch to batch variations in EL spectra and dispersion of CIE coordinates in control devices (Fig. 3e and Supplementary Fig. 23a), ascribed to nonequilibrium crystal growth and hence uncontrollable local film composition. In contrast, EL spectra and CIE coordinates of VAC-treated devices are highly reproducible between batches, resulting from self-moderated halide rearrangement during the VAC-treatment (Fig. 3e and Supplementary Fig. 23b). When comparing the devices processed with different duration of VAC treatment, we observe a remarkable EQE enhancement in one minute of treatment, that is, with averaged peak EQE values improving from ~0.6% to ~3.8% (Fig. 3f), which well corresponds to the dramatical morphological variations in the same time scale from SEM results (Supplementary Fig. 20). We believe that perovskite re-crystallization, enlarged grain size and improved local homogeneity collectively help to reduce the defect density and hence reduce non-radiative recombination. In addition, the isolated nano-structures may also contribute to the efficiency improvement due to enhanced light-out coupling^3^. With increasing the processing duration, the EQE values gradually approach saturation. We assign this to a slow diffusion-mediated defect healing process from the gradually improved homogeneity that reduces local lattice mismatch and strain-induced interfacial defects^42,43^. Notably, one minute of VAC-treatment is sufficient for improving the efficiency but not the spectral stability (Fig. 3e), indicating that the discontinuous morphology has little impact on improving phase stability. In other words, a large perovskite grain with size scale of hundreds of nanometres in our samples can hardly be the reason for the suppression of phase segregation within the grain, as probed in previous reports showing that the phase segregated domain can be as small as ~8 nm^30,32^. We also notice that the devices with five-minute treatment show comparable colour stability to those with twenty-minute treatment (Fig. 3e), corresponding well to the time scale of the disappearance of high-energy phases as observed in Fig. 3c. It further confirms the critical role of high compositional homogeneity in improving phase stability.

Given the critical role of the diffusive environment on retarding crystallization for halide rearrangement, a proper solubility of perovskite precursors in the solvent vapour might be the key to achieving high compositional homogeneity. We thus perform additional experiments using dimethyl sulfoxide (DMSO) and chloroform as the alternative vapours to further understand the VAC treatment. DMSO is another commonly used solvent for perovskite precursors, while chloroform is a well-known “anti-solvent” that is widely used to accelerate perovskite crystallization^44^. Considering that the vapour residues in the glovebox may affect the results, we also prepare the samples without introducing any vapour on purpose, that is, leaving the as-casted films in the glovebox for the same duration. As shown in Supplementary Fig. 24, the introduction of chloroform vapour has no positive effect on either device efficiency or spectral stability, which can be attributed to the poor solubility of perovskite precursors in chloroform, leading to a fast crystallization and freezing of the composition, and hence resulting in high heterogeneity. In contrast, DMSO treatment gives comparable improvement as DMF vapour, further rationalising our understanding of the effect of the vapour treatment.

### The general applicability of VAC-treatment and device optimization

We proceed to explore the VAC-treatment in other material systems, aiming to further improve the device performance and validate the general applicability. We incorporate a small amount of rubidium ions (Rb^+^) in our perovskites, that is, using a precursor composition of Rb^+^: Cs^+^: FA^+^: Pb^2+^: [Br_0.6_ + Cl_0.4_]^−^ = 0.1: 1.2: 0.2: 1: 3.5. Consistent with the previous reports in perovskite solar cells^45,46^, the incorporation of Rb^+^ effectively suppresses non-radiative recombination as indicated by a considerable enhancement of peak external PLQY (25%) and a prolonged PL lifetime (Supplementary Figs. 25a, b). The small amount of Rb^+^ addition has little impact on the film morphology (Supplementary Fig. 25c).

**Fig. 4:**
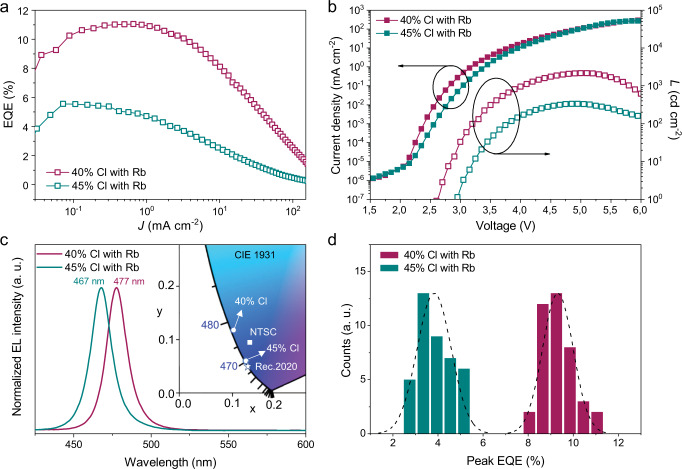
The device performance of Rb-passivated perovskites with 40% and 45% Cl contents. **a** EQE-current density (*J*) curves (*J*-EQE). **b** Current density–voltage–luminance (*J–V–L*) characteristics. **c** EL spectra and CIE colour coordinates. The square and pentagram in the CIE 1931 (x, y) chromaticity diagram represent the colour coordinates of primary blue specified in the National Television System Committee (NTSC) and recommendation bt.2020 (Rec.2020), respectively. **d** Histograms of the peak EQEs extracted from 40 devices for each case.

We show the characteristics of the best-performing VAC-treated Rb-device using 40% Cl content in Fig. 4. The device exhibits blue emission peaking at 477 nm with FWHM of 18 nm. The corresponding CIE coordinates are (0.107, 0.115), approaching the primary blue (0.14, 0.08) specified by the National Television System Committee (NTSC). Compared to the device without using VAC-treatment (Supplementary Fig. 26), the treated device shows a significant enhancement of EQE value up to 11.0%. The luminance rises rapidly after the device turns on at a low voltage of 2.6 V, reaching a peak value of 2,180 cd cm^−2^ at 5.0 V (106 mA cm^−2^). The low turn-on voltage and high brightness indicate efficient charge injection, which is usually very challenging in strongly confined perovskites^14^. We observe no peak shift during voltage scans until reaching a high bias at 6.0 V (~300 mA cm^−2^) (Supplementary Fig. 27a), analogous to the device without Rb^+^ incorporation, further indicating that phase segregation in VAC-treated devices is mainly mediated by the device damage at harsh operating conditions. In addition, we demonstrate that no EL shift can be observed even after 75 min of operation at 3 V (~0.1 mA cm^−2^, with initial luminance of ~10 cd m^2^) (Supplementary Fig. 27b). Although Rb^+^ addition significantly improves the device efficiency, we have not observed any distinct effect on operational stability (~3 min, Supplementary Fig. 27c, d). The short operational lifetime could be a result of Joule heating and ion-migration induced material and/or interfacial degradation under the bias^9,28^, as well as Al diffusion and relevant redox reaction between Pb^2+^ and Al^0^^47^. An EQE histogram for 40 devices shows an average peak EQE of 9.3% with a low standard deviation of 0.67%, indicating high reproducibility of the VAC-treatment.

Further increasing Cl content to 45% results in deep-blue emission, whose device characteristics are also summarized in Fig. 4. The corresponding CIE coordinates are (0.130, 0.059), very close to Rec. 2020 specified blue standards (0.131, 0.046). The deep-blue PeLEDs achieves a peak EQE of 5.5% and an average peak EQE of 3.9% with a standard deviation of 0.76%, which are among the best for PeLEDs with ideal deep-blue emission.

We demonstrate that the VAC-treatment is also applicable for improving the colour stability and device performance of low-dimensional perovskites with mixed bromide/chloride anions, e.g. the typical phenethylammonium (PEA^+^)-modified CsPb(Br_0.7_Cl_0.3_)_3_ (Supplementary Fig. 28). These results indicate that the wavelength of the previously reported high-performance sky-blue PeLEDs based on quasi-2D perovskites could be pushed to a bluer region without any negative impacts on their colour stability and device efficiency.

In summary, we have demonstrated that the notorious colour instability issues in mixed halide blue PeLEDs can be substantially mitigated across a wide range of emission colours from sky blue to deep blue region (490–451 nm). The excellent phase stability is mainly achieved by the development of a vapour-assisted crystallization technique that effectively suppresses the ion migration and compositional heterogeneity. Particularly, for the first time, we show high-efficiency and spectrally stable blue and deep-blue PeLEDs based on mixed halide 3D perovskites, with respective peak EQEs of 11.0% and 5.5%, presenting two of the most efficient blue PeLEDs to date. Our findings are also applicable to the prevailing low-dimensional blue perovskite emitters, indicating a bright future for further improvement of blue PeLEDs by combining these two strategies. Our research thus provides a broad avenue for future development of blue perovskite emitters, representing another milestone towards practical implementation of perovskite light-emitting diodes in full-colour displays and lighting applications. Beyond that, stabilized mixed halide perovskites are also of great interest for a wide range of perovskite applications where the bandgap needs to be finely controlled, for instance, lasing and tandem solar cells.

## Methods

### Materials

Caesium bromide (CsBr, 99.999%), lead bromide (PbBr_2_, 99.999%), lead chloride (PbCl_2_, 99.999%) was purchased from Alfa Aesar. Formamidinium bromide (FABr) and phenethylammonium bromide (PEABr) were purchased from Greatcell Solar. Rubidium bromide (RbBr, 99.99%), polyvinylpyridine (PVP, average Mw ~55,000), 4,7,10-trioxa-1,13-tridecanediamin (TTDDA), poly(9-vinylcarbazole) (PVK, average Mn 25,000–50,000) were purchased from Sigma Aldrich. The NiO_x_ nano-crystals were purchased from Avantama AG and were used without additional treatment. 1,3,5-tris(1-phenyl-1H-benzimidazol-2-yl)benzene (TPBi) was purchased from Luminescence Technology corp. Other materials for device fabrication were all purchased from Sigma-Aldrich.

### Preparation of the perovskite solution

Perovskite precursors (CsBr: FABr: PbBr_2_: PbCl_2_: TTDDA) with a molar ratio of 1.2: 0.3: x*:* y: 0.1 (where x + y = 1) were mixed and dissolved in dimethyl sulfoxide (DMSO). The precursor concentration as determined by Pb^2+^ is 0.15 M for 30–40% Cl, 0.13 M for 45% Cl, 0.11 M for 50% Cl, and 0.09 M for 57% Cl, respectively. The precursor solutions were stirred at 80 °C for 4 h before use. For the low-dimensional perovskites, precursors (PEABr: CsBr PbBr_2_: PbCl_2_) with a molar ratio of 0.9:1.1:0.4:0.6 mixed and dissolved in DMSO to make a solution with 30% Cl-content. The precursor concentration determined by Pb^2+^ is 0.15 M.

### PeLED fabrication

Glass substrates with patterned Indium tin oxide (ITO) were sequentially cleaned by detergent and TL-1 (a mixture of water, ammonia (25%) and hydrogen peroxide (28%) (5:1:1 by volume)). The clean substrates were then treated by ultraviolet-ozone for 10 min. NiO_x_ was spin-coated in air at 4000 r.p.m. for 30 s, followed by baking at 150 °C for 10 min in air. The substrates were then transferred into a nitrogen-filled glovebox (<0.1 ppm H_2_O, < 0.1 ppm O_2_). PVK (4 mg mL^−1^ in chlorobenzene) was deposited at 3000 r.p.m. followed by thermal annealing at 150 °C for 10 min. Next, a thin layer of PVP (2.0 mg mL^−1^ in isopropyl alcohol (IPA)) was deposited at 3000 r.p.m. and baked at 100 °C for 5 min. After cooling down to room temperature, the perovskite solutions with varying bromide/chloride ratios were deposited at 3000 r.p.m. Directly after spin-coating, the films were put in an unsealed ⌀60 mm petri-dish (with lid) at room temperature, where 20 µl of dimethylformamide had been dropped 10 min prior to the film placement. After 20 min of vapour assisted crystallisation (VAC) treatment, the films were annealed at 80 °C for 8 min. For low-dimensional perovskite films with mixed halides, the treatment duration is 10 min and the annealing condition is 80 °C for 5 min. Finally, the electron transport layer TPBi and top contacts LiF/Al (1 nm / 100 nm) were deposited by thermal evaporation through shadow masks at a base pressure of ~10^−7^ torr. The device structure for single hole devices in Supplementary Fig. 6 is ITO/NiO_x_ or not/PVK/molybdenum oxide (MoO_3_) (7 nm)/Au. The device area was 7.25 mm^−2^.

### PeLED characterization

All PeLED device characterizations were performed at room temperature in a nitrogen-filled glovebox without encapsulation. A Keithley 2400 source-meter and a fibre integration sphere (FOIS-1) coupled with a QE Pro spectrometer (Ocean Optics) were utilized. The absolute radiance was calibrated by a standard Vis–NIR light source (HL-3P-INT-CAL plus, Ocean Optics). The PeLED devices were measured on top of the integration sphere and only forward light emission can be collected. The devices were swept from zero bias to forward bias with a step voltage of 0.05 V, lasting for 100 ms at each voltage step for stabilisation. The sweep duration from 1 to 7 V is 70 sec (with a scan rate of 86 mV S^−1^). The EQE and spectral evolution with time was measured using the same system.

### Perovskite film characterization

Top-view scanning electron microscope (SEM) images were tested by LEO 1550 Gemini. Steady-state PL spectra of the perovskite films were recorded by a fluorescent spectrophotometer (F-4600, HITACHI) with a 200 W Xe lamp as an excitation source. UV–Vis absorbance spectra were collected using a PerkinElmer model Lambda 900. X-ray diffraction patterns were measured using a Panalytical X’Pert Pro with an X-ray tube (Cu Kα, λ = 1.5406 Å).

X-ray photoelectron spectroscopy (XPS) tests were performed by a Scienta ESCA 200 spectrometer in ultrahigh vacuum (~1 × 10^−10^ mbar) with a monochromatic Al (Kɑ) X-ray source providing photons with 1,486.6 eV. The experimental was set so that the full-width at half-maximum of clean Au 4f 7/2 line (at the binding energy of 84.00 eV) was 0.65 eV. All spectra were characterized at a photoelectron take-off angle of 0°. Ultraviolet photoelectron spectroscopy (UPS) was carried out using a Kratos AXIS Supra on perovskite samples spun-cast on ITO/NiO_x_/PVK/PVP. He I (21.22 eV) radiation was generated from a helium discharge lamp. Samples were biased at 9.1 V.

In-situ PL of the crystallisation process was collected using the integrating sphere and the QE Pro spectrometer as described above, and a 365 nm UV laser as excitation source. In-situ transmittance tests were performed using the same spectrometer but with a solar simulator (AM 1.5G) as the light source. A ND filter was used to decrease the light intensity. The systems were illustrated in Supplementary Fig. 21.

Time-correlated single photon counting (TCSPC) measurements were carried out by using an Edinburgh Instruments FL1000 with a 405 nm pulsed picosecond laser (EPL-405). Fluence-dependent PLQY was measured using a 405 nm continuous wave laser, an integrating sphere and the same spectrometer. The perovskite films were deposited on ITO/NiO_x_/PVK/PVP substrates under identical conditions as for the PeLEDs, and encapsulated using glass slides and UV-curable resin.

Grazing-incidence wide-angle X-ray scattering (GIWAXS) was recorded in Shanghai Synchrotron Radiation Facility. The diffraction patterns were collected by two dimensional MarCCD 225 detector with 234 mm from samples to the detector. All the samples were protected with N_2_ gas during the measurements. To assure the diffraction intensity, an exposure time of 15 s was adopted with an incidence angle of 0.5°, and the wavelength of the X-ray was 1.24 Å (10 KeV). For all these tests, the perovskite films were deposited on ITO/NiO_x_/PVK/PVP substrates under identical conditions as device fabrication.

### Scanning transmission electron microscopy (STEM) and energy-dispersive X-ray spectroscopy (EDX)

The STEM samples were fabricated by using the FEI Focused Ion Beam (FIB) system (Helios Nanolab 600i). A FEI Titan-G2 Cs-corrected transmission electron microscope with 300 KV accelerating voltage was used to get the high angle angular dark field (HAADF) images of the samples. The STEM elemental mapping images were collected by four silicon drift windowless detectors (Super-EDX) in the FEI Titan-G2 Cs-corrected transmission electron microscope. The energy resolution of the Super-EDX was 137 eV.

### Transient absorption

A femtosecond oscillator (Mai Tai, Spectra Physics) is used as a seed laser for a regenerative amplifier (Spitfire XP Pro, Spectra Physics) which generates well collimated beam of femtosecond pulses (800 nm, 80 fs pulse duration, 1 kHz repetition rate). The second harmonic generated by a BBO crystal was used as pump (400 nm). White light continuum (WLC) as the probe was produced by focusing the 800 nm fs pulse on a thin CaF_2_ plate. Polarization between the pump and probe was set to the magic angle (54.7°). Both pump and probe pulses are monitored to compensate for the laser fluctuations during the measurements.

### Admittance spectroscopy

For the defect studies, we used a setup consisting of a Zurich Instruments MFLI lock-in amplifier with MF-IA and MF-MD options, a Keysight Technologies 33600A function generator and a cryo probe station Janis ST500 with a Lakeshore 336 temperature controller. For determining the ion signature using admittance spectroscopy, we varied the sample temperature from 200 K to 350 K in 5 K steps, controlled accurately within 0.01 K and using liquid nitrogen for cooling. The capacitance in term of a C || R equivalence model was measured by applying an ac voltage with amplitude of *V*_ac_ = 20 mV and varying the angular frequency from 0.6 Hz to 3.2 MHz. The rates *e*_t_ are obtained from the peak maxima of the derivative of the capacitance. These rates are linked to the diffusion coefficient *D* in terms of the underlying hopping process of the mobile ions^48,49^,

1 $$e_{\mathrm{t}} = \frac{{e^2N_{{\mathrm{eff}}}D}}{{k_{\mathrm{B}}T\varepsilon _0\varepsilon _{\mathrm{R}}}},$$

where *N*_eff_ refers to the effective doping density, *e* is the elementary charge, *k*_B_ is the Boltzmann constant, *T* the temperature, *ε*_0_ the dielectric constant and *ε*_R_ the relative permittivity. For the calculation of *D*_300K_, we used a dielectric permittivity of 19.2^50^. Since ion migration is a thermally activated process, the diffusion coefficient depends on the temperature,

2 $$D = D_0exp\left( { - \frac{{E_A}}{{k_BT}}} \right)$$

with the activation energy for ion migration *E*_A_ and the diffusion coefficient at infinite temperatures *D*_0_. Subsequently, *E*_A_ and *D*_0_ can be extracted from the slope and the cross section with the emission rate axis using Eqs. (1) and (2). By taking into account the surface polarization caused by the accumulation of mobile ions at the interfaces of the perovskite layer, the ion concentration *N*_i_ is determined as^51^,

3 $$N_{\mathrm{i}} = \frac{{k_{\mathrm{B}}T{\Delta}C^2}}{{e^2\varepsilon _0\varepsilon _{\mathrm{R}}}}$$

Here, ΔC refers to the capacitance step in the admittance spectra of the contributing ions.

## Supplementary information

Supplementary Information

## Data Availability

The data that support the plots within this paper and other findings of this study are available from the corresponding author upon reasonable request.
