# Supplementary material for: Mixed halide perovskites for spectrally stable and high-efficiency blue light-emitting diodes
Source: Nat Commun. 2021 Jan 13;12:361. doi: 10.1038/s41467-020-20582-6 (PMC7806600; doi:10.1038/s41467-020-20582-6)
Supplement: Supplementary file 1 — Supplementary Information [file 41467_2020_20582_MOESM1_ESM.pdf]

Mixed Halide Perovskites for Spectrally Stable and High-Efficiency Blue Light-Emitting Diodes

Max Karlsson[1,#], Ziyue Yi[1,2,#], Sebastian Reichert[3], Xiyu Luo[1,4], Weihua Lin[5], Zeyu Zhang[6], Chunxiong Bao[1], Rui Zhang[1], Sai Bai[1], Guanhaojie Zheng[1], Pengpeng Teng[1], Lian Duan[4], Yue Lu[6], Kaibo Zheng[5,7], Tönu Pullerits[5], Carsten Deibel[3], Weidong Xu[1]*, Richard Friend[2], and Feng Gao[1]*

[1]Department of Physics, Chemistry and Biology (IFM), Linköping University, Linköping, Sweden.

[2]Cavendish Laboratory, University of Cambridge, Cambridge, UK.

[3]Institut für Physik, Technische Universität Chemnitz, Chemnitz, Germany.

[4]Key Lab of Organic Optoelectronics and Molecular Engineering of Ministry of Education, Department of Chemistry, Tsinghua University Beijing, China.

[5]Chemical Physics and NanoLund, Lund University, Lund, Sweden.

[6]Institute of Microstructure and Properties of Advanced Materials, Beijing University of Technology, Beijing, China.

[7]Department of Chemistry, Technical University of Denmark, DK-2800 Kongens Lyngby, Denmark.

[#]These authors contributed equally: M. K. and Z. Y.

*These authors jointly supervised this work: W.X. (weidong.xu@liu.se) and F. G (feng.gao@liu.se).

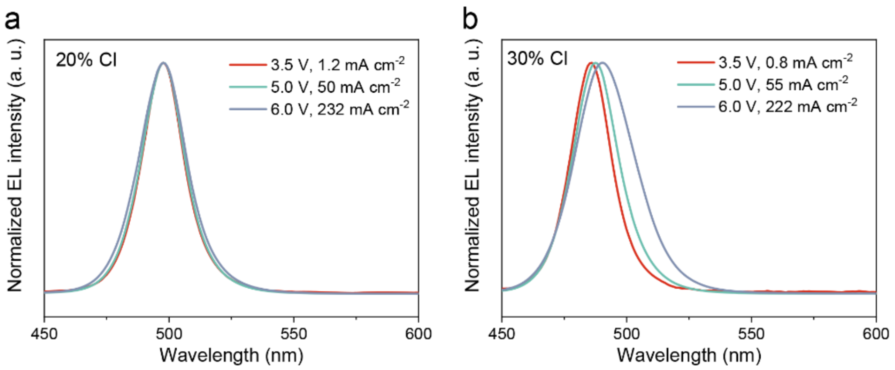

Supplementary Figure 1. Colour stability of bromide/chloride mixed halide PeLEDs dependent on chloride content. a, EL spectra of 20% Cl under different operational conditions. **b**, EL spectra of 30% Cl under different operational conditions. The devices here were prepared by annealing directly after spin-casting.

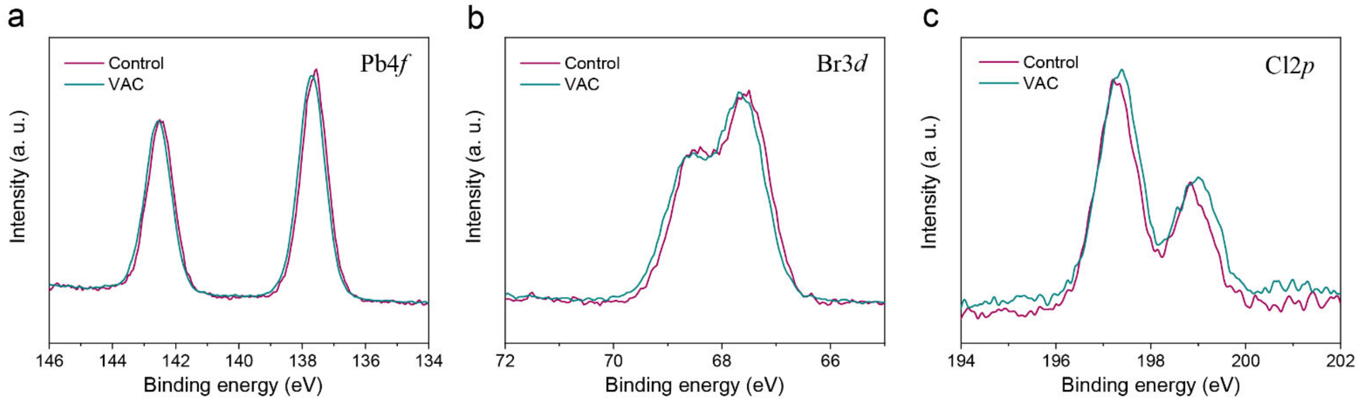

Supplementary Figure 2. Determination of the content of Br and Cl anions in the thin films by XPS for control and VAC films (40% Cl addition). a, Pb4*f*; **b**, Br3*d*. **c**, Cl2*p* core level spectra. All the samples were prepared on ITO/NiO_x/PVK/PVP substrates. The respective stoichiometric ratio of Br and Cl is referenced to the Pb4*f* intensity. We observe no obvious difference in element ratio between the VAC- and control samples. The content of Cl anions in total halides for both cases is 42%. $[Br + Cl]^-$: Pb^{2+} is equal to 3.2.

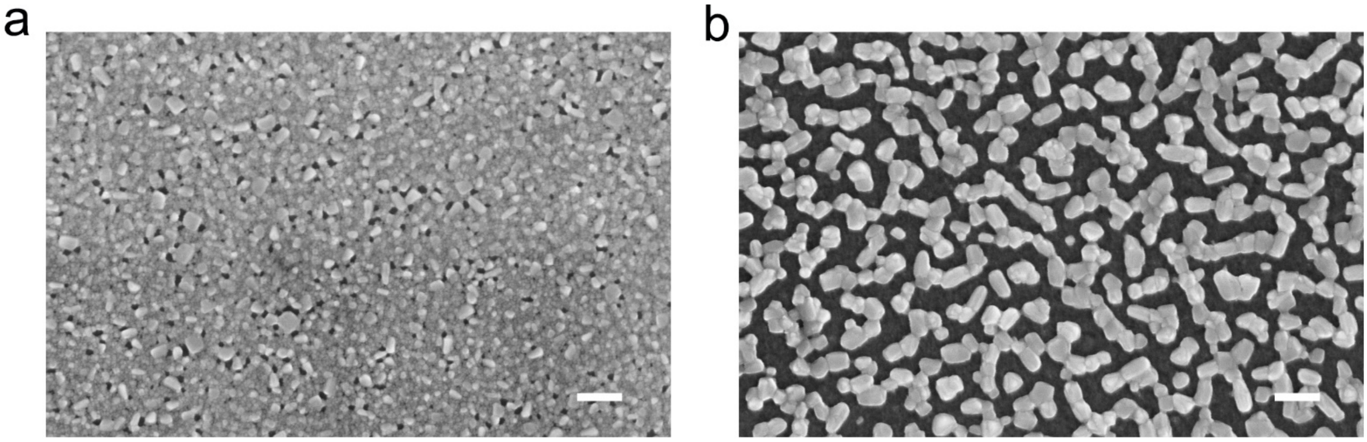

Supplementary Figure 3. Top-view SEM images for the perovskite films. a, Control film. **b**, VAC-film. The perovskite films are deposited on ITO/NiO_x/PVK/PVP. The scale bars are 400 nm.

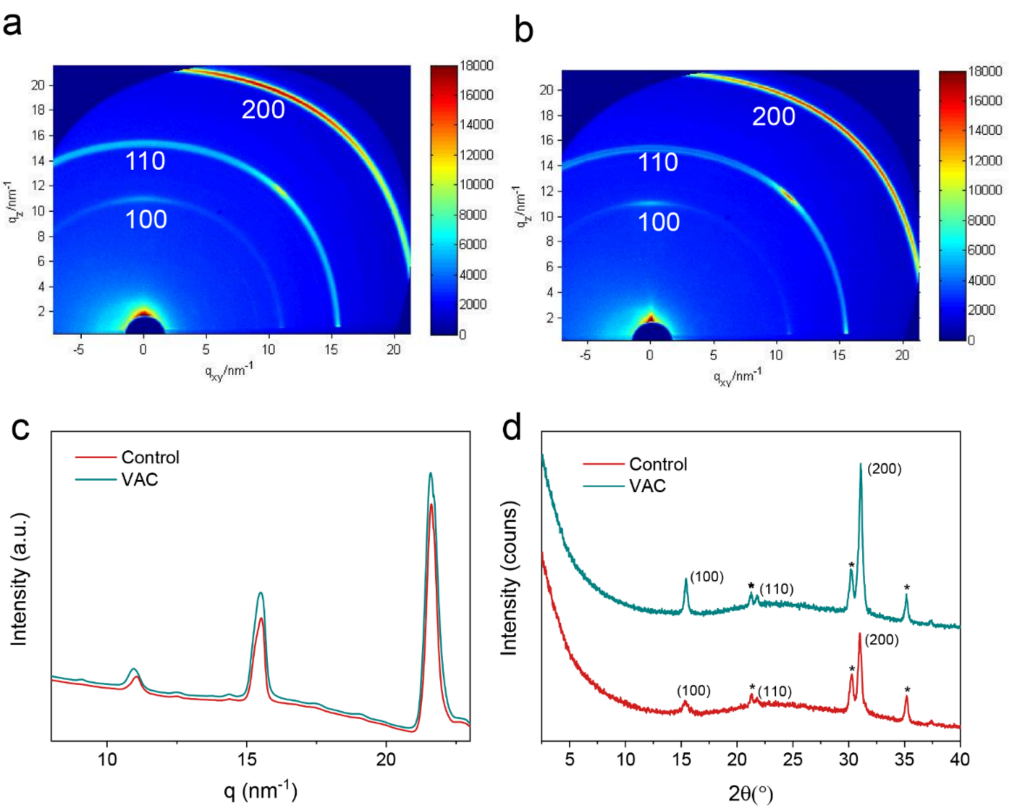

Supplementary Figure 4. Crystallographic data for films with and without treatment. a, **b**, GIWAXS patterns for control (**a**) and VAC-treated film (**b**). **c**, 2D-GIWAXS plot extracted by integrating over all angles. **d**, XRD patterns. Asterisks mark ITO-peaks.

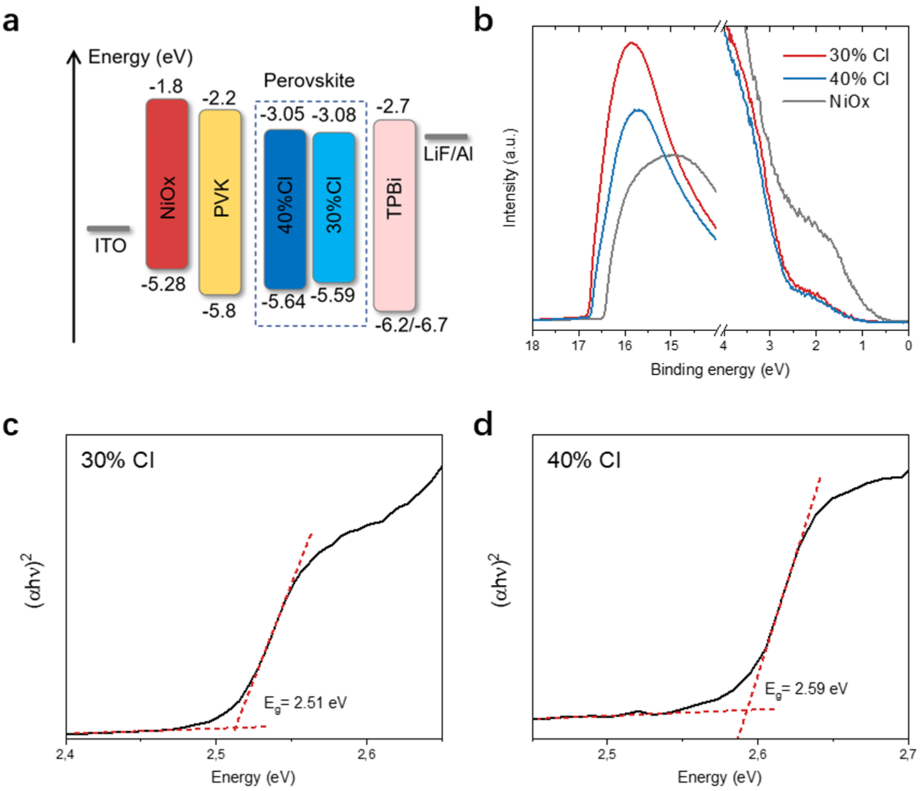

Supplementary Figure 5. Band energy landscape. a, Energy level diagram of charge transport layers and perovskites with different Cl-content. **b**, Ultraviolet photoelectron spectroscopy (UPS) spectra of NiO_x and perovskites with 30% and 40% Cl-content. **c, d,** Tauc-plots of perovskites with 30% and 40% Cl-content. The valence band (VB) of perovskites and NiO_x are determined by UPS results. The conduction band (CB) of perovskites are calculated from the VB and the optical bandgap determined by Tauc-plots. Other energy levels are taken from literature[1-4].

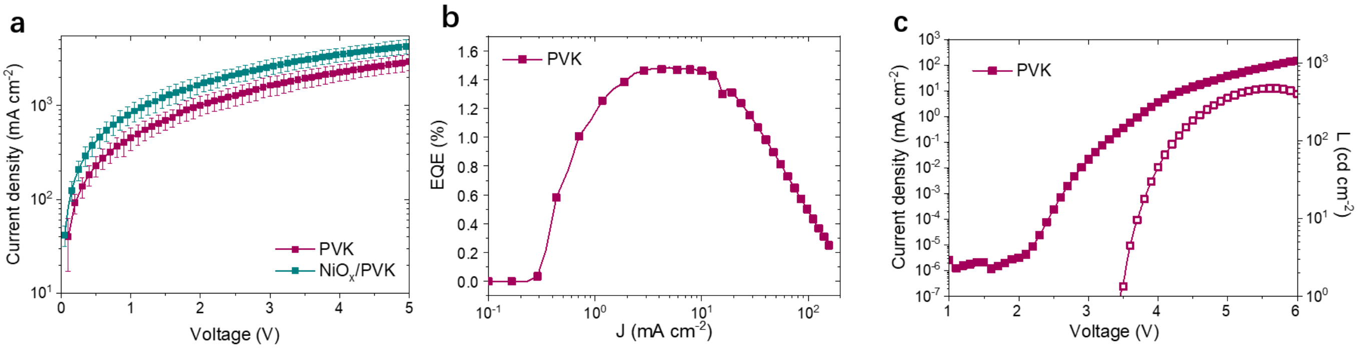

Supplementary Figure 6. Impact of the cascade energy alignment of hole transport layers on hole injection. a, *J-V* characteristics of single-carrier hole-only devices with and without NiO_x depicting the improved hole injection. **b, c,** Performance of representative 40% Cl-devices with only PVK as the hole injection layer: EQE as a function of current density (*J-EQE*) (**b**); Current density and luminance as a function of voltage (*J-V-L*) (**c**).

The high charge carrier mobility of TPBi and large hole injection barrier caused by the deep lying valence band of blue perovskites give rise to imbalanced charge injection. For this reason, we employed a combination of hole transport materials (NiO_x and PVK) to construct a cascaded energy level alignment (Supplementary Fig. 5). The facilitated hole injection is evident from single-carrier hole-only devices (Supplementary Fig. 6a). The much-improved device performance with NiO_x/PVK (Fig. 1c and Supplementary Fig. 10) bi-layers and lower turn-on voltage compared to that with only PVK (Supplementary Fig. 6b and c) indicate a more balanced charge injection.

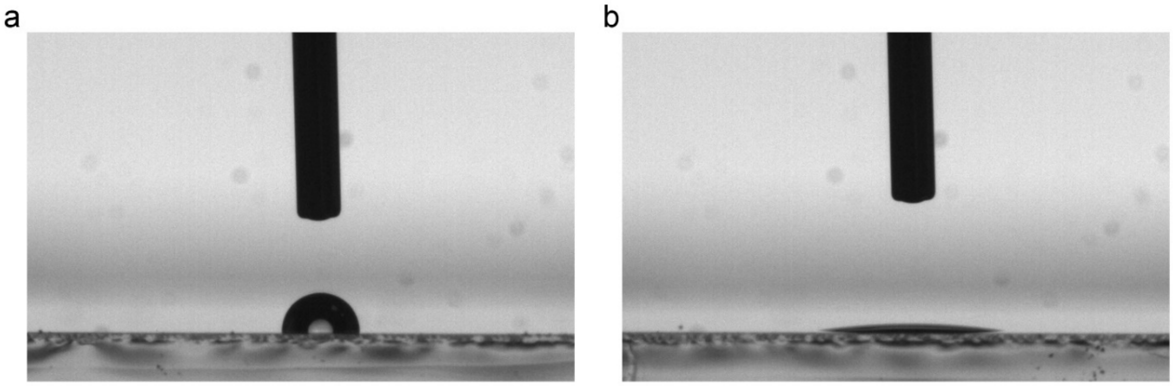

Supplementary Figure 7. Water contact angle on different surfaces. a, ITO/NiO_x/PVK. **b,** ITO/NiO_x/PVK/PVP. The coating condition is identical to device fabrication. The deduced contact angle is ~95° on the ITO/NiO_x/PVK surface and ~10° on the ITO/NiO_x/PVK/PVP surface, respectively. These results suggest a change from hydrophobic to hydrophilic surface after PVP deposition.

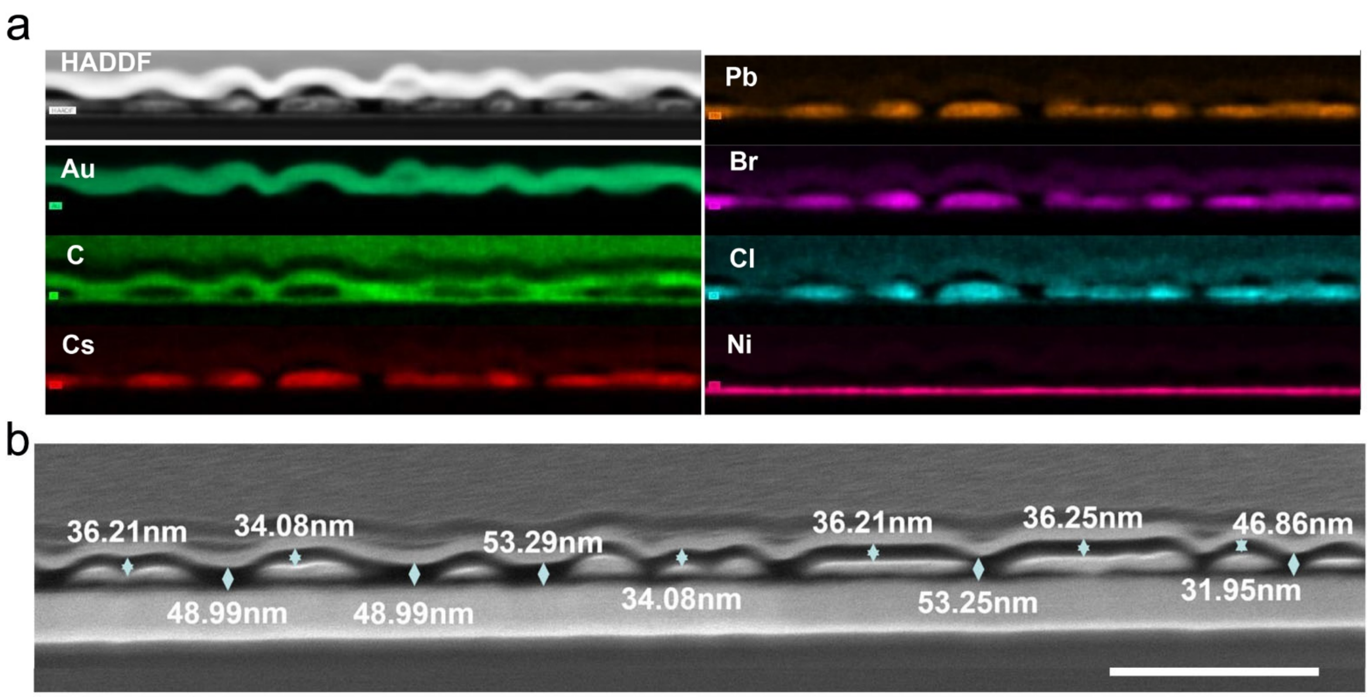

Supplementary Figure 8. Morphology. a, a HAADF cross-sectional image and corresponding EDX mapping images for various elements. **b**, a HAADF cross-sectional image showing that the thickness of the organic layer is different on the hole injection layer compared with that on perovskite grains. The thickness of both PVK and NiO_x layers are ~10 nm. The scale bar is 500 nm.

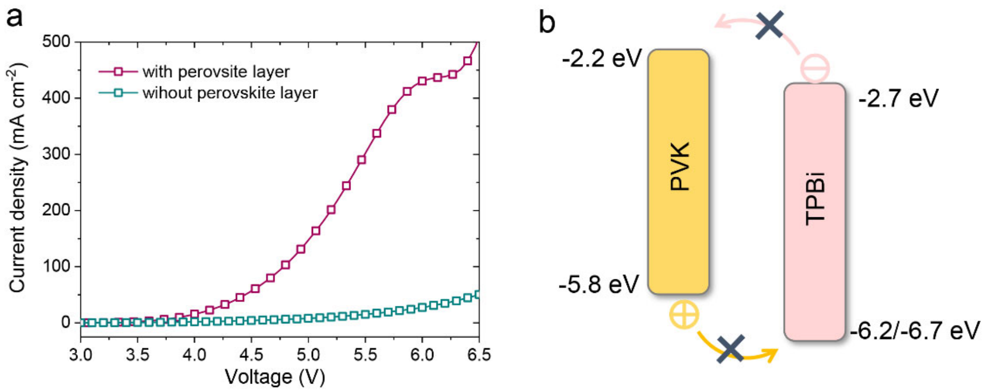

Supplementary Figure 9. Explanation for low leakage current in VAC-treated devices. a, *J-V* characteristics for devices with and without the perovskite layer. **b**, Energy diagrams for PVK/TPBi. The energetic levels of PVK and TPBi are taken from the literature[1-3].

Given the fact that our PeLEDs show decent device performance, we believe the discontinuous morphology in VAC-treated films does not necessarily lead to strong leakage currents under normal operational conditions. Probably it is because the organic layer the bottom hole injection layer is much thicker (~50 nm) than that on top of perovskite grains (~35 nm), as suggested by HAADF and carbon distribution from EDX device cross-sectional images (Supplementary Figure 8). The uneven layer thickness of TPBi helps to increase the local resistance at the PVK/TPBi interface, hindering leakage current injection. In addition, the large injection barrier caused by the energy level mismatch between TPBi and PVK could be another important reason (Supplementary Figure 9b). Following these considerations, we prepared the devices without a perovskite layer (Supplementary Figure 9a). It is apparent that current injection between transport layers is unfavourable at normal working conditions of our LEDs, and the current density (e.g. ~25 mA cm^{-2} at 6 V) is much lower than the devices with perovskites. We notice that a similar morphology is also visible in other high-performance PeLEDs[5,6], as well as in our Rb-incorporated devices (Supplementary Figure 25).

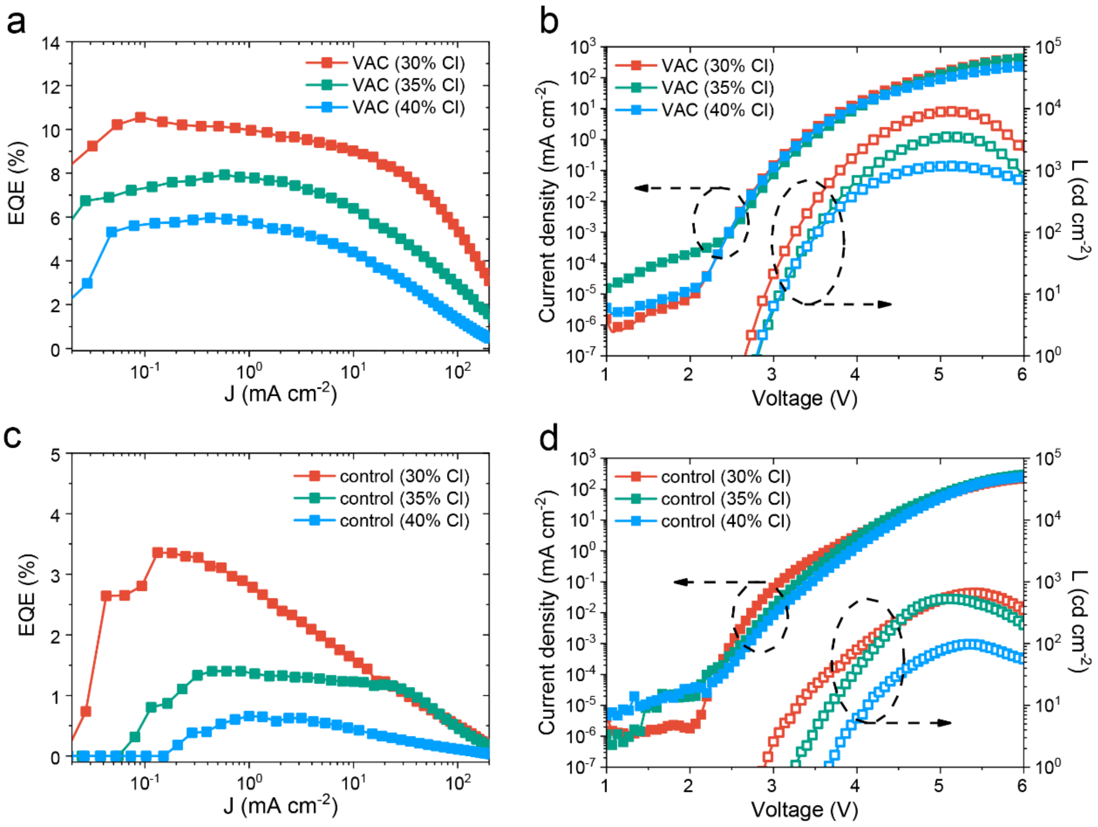

Supplementary Figure 10. Performance of representative devices prepared from precursors with the stoichiometry of Cs^+: FA^+: Pb^{2+}: $[Br_{1-x}+Cl_x]^-$: TTDDA= 1.2: 0.3: 1: 3.5: 0.1 (x = 30%, 35%, 40%). a, b, VAC-treated devices: *J-EQE* (**a**); *J-V-L* (**b**). **c**, **d**, control devices: *J-EQE* (**c**); *J-V-L* (**d**).

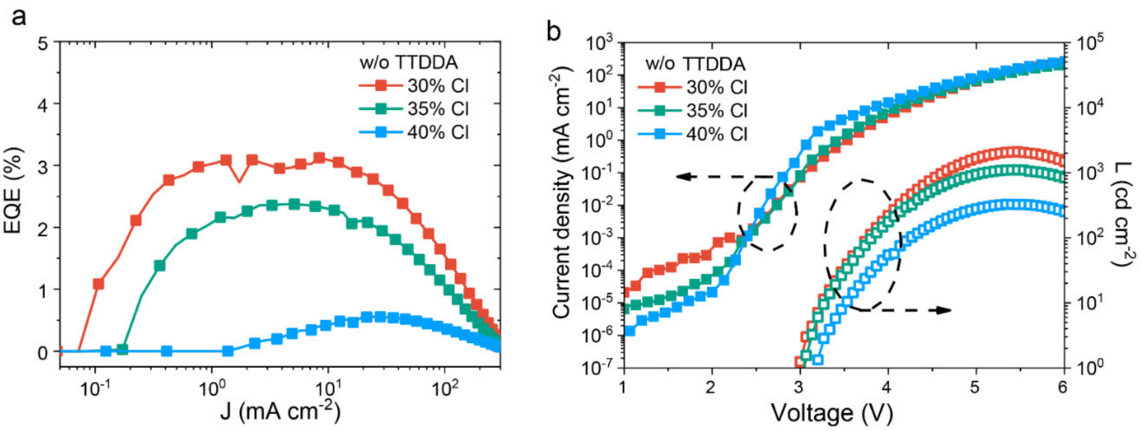

Supplementary Figure 11. Performance of representative VAC-treated devices prepared from precursors without TTDDA addition. a. *J-EQE*. **b.** *J-V-L*. The stoichiometry of Cs^+: FA^+: Pb^{2+}: $[Br_{1-x}+Cl_x]^-$ is equal to 1.2: 0.3: 1: 3.5 (x = 30%, 35%, 40%).

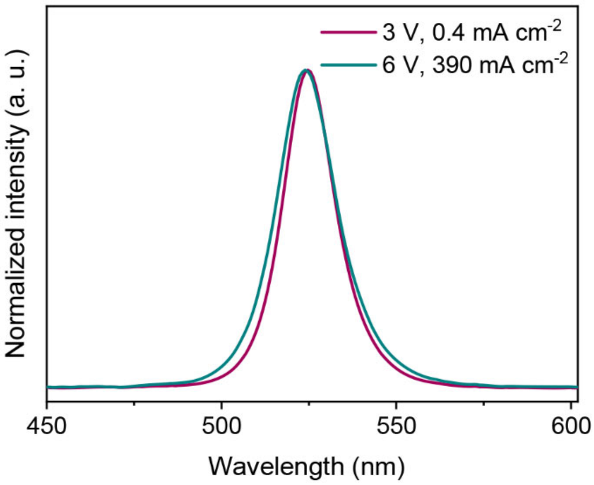

Supplementary Figure 12. EL spectra of Br-perovskites at low and high voltage/current density. The perovskite layers are prepared from a precursor stoichiometry of Cs^{+}: FA^{+}: Pb^{2+}: Br^{-} = 1.2: 0.3: 1: 3.5. The Pb^{2+} concentration is 0.15 M.

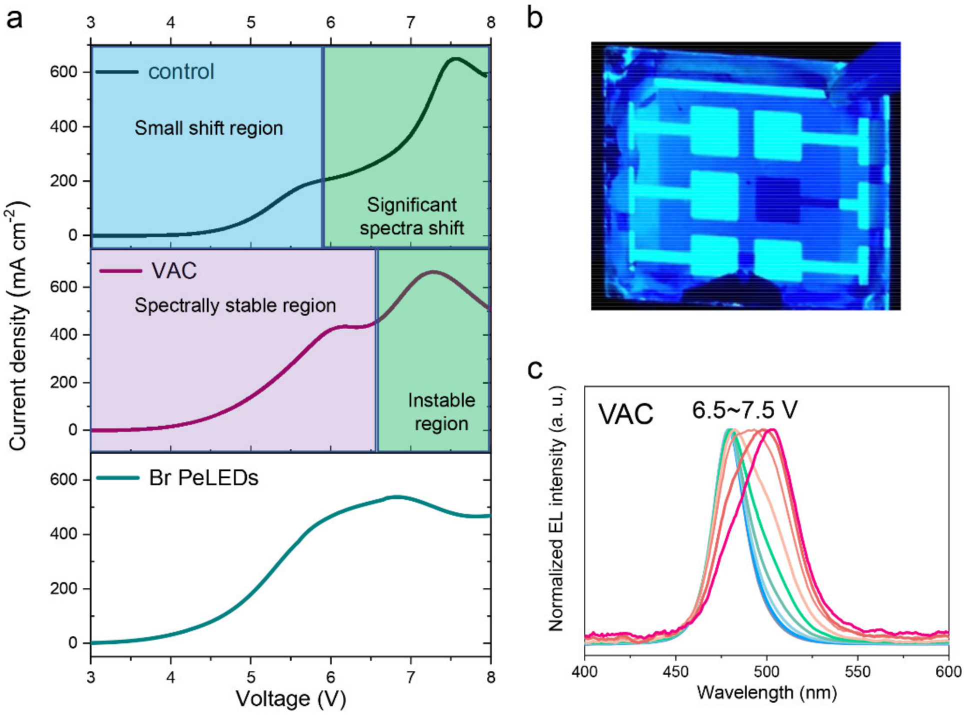

Supplementary Figure 13. Understanding the spectral shift of VAC-treated devices at harsh operational conditions. a, *J*-*V* characteristics of the control, VAC-treated blue PeLEDs, and a Br-based green PeLED. All the *J*-*V* characteristics show plateau-like features when the voltage approaches 6 V, suggesting that the spectra shifts of VAC-devices are triggered by device damage. **b**, A photograph of a VAC-treated device under UV-light. The dark pixel was swept from 1 to 6.5 V with a scan rate of 86 mV S^{-1} (~64 seconds in total). **c**, The EL spectral evaluation during a voltage scan above 6.5 V.

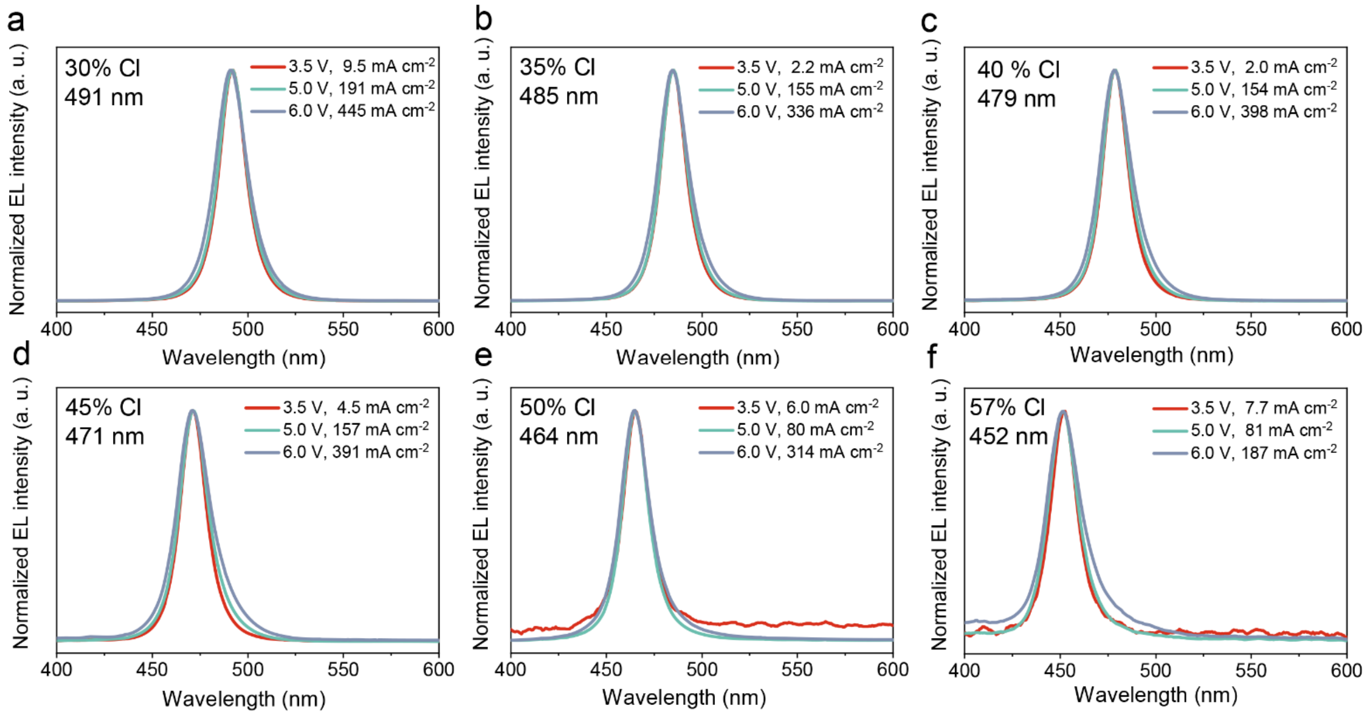

Supplementary Figure 14. EL spectral evolution during voltage sweep for the VAC-treated devices with varying chloride content (30~57%). **a**, 30% Cl. **b**, 35% Cl. **c**, 40% Cl. **d,** 45% Cl. **e**, 50% Cl. **f**, 57% Cl. The precursor concentration as determined by Pb^{2+} is 0.15 M for 30~40% Cl, 0.13 M for 45% Cl, 0.11 M for 50% Cl, and 0.09 M for 57% Cl, respectively. All the devices show maximum brightness around ~5.0 V.

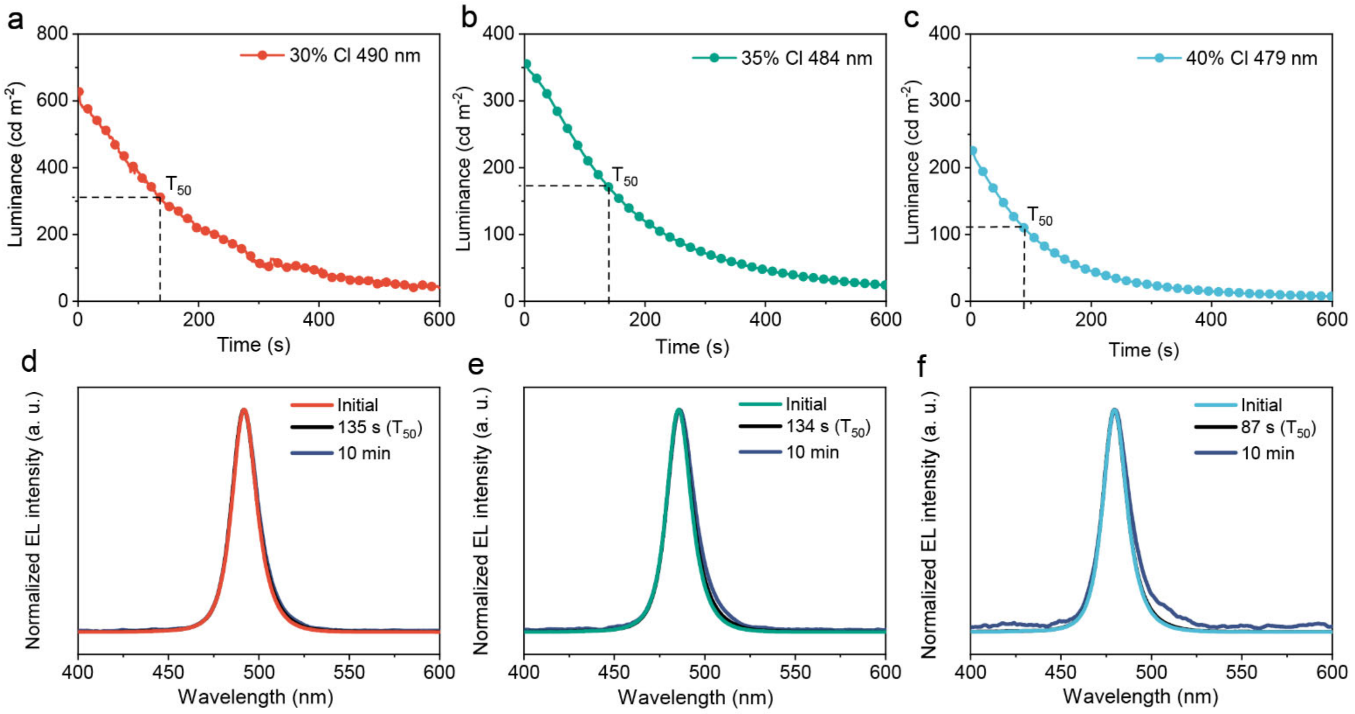

Supplementary Figure 15. Spectral and operational stability for optimized VAC-treated devices with varying chloride content (30%, 35%, 40%). a-c, Operational stability under constant current of 5 mA cm^{-2} for 30%- (**a**), 35%- (**b**) and 40% (**c**)-Cl devices. **e-f**, Normalized EL spectra for 30%- (**d**), 35%- (**e**) and 40% (**f**)-Cl devices extracted at initial, at T_{50} and after 10 minutes. The T_{50} lifetime is 135 s, 134 s and 87 s for 30%, 35%, and 40%-Cl device, respectively.

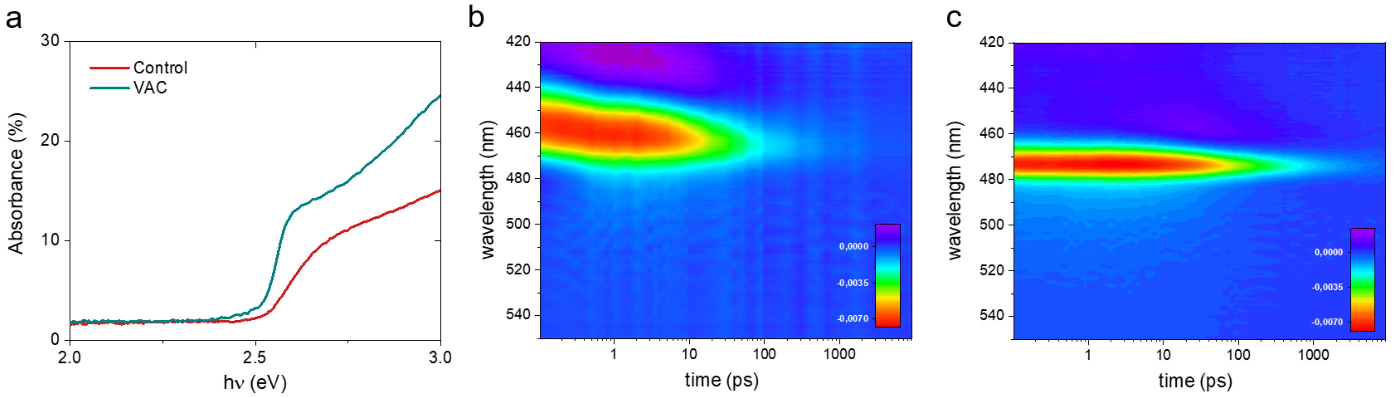

Supplementary Figure 16. Impact of VAC-treatment on perovskite homogeneity. a, Absorbance. **b, c,** Time-resolved transient absorption spectra of the control film (**b**) and treated film (**c**). The excitation power is around 25 mW cm^{-2}.

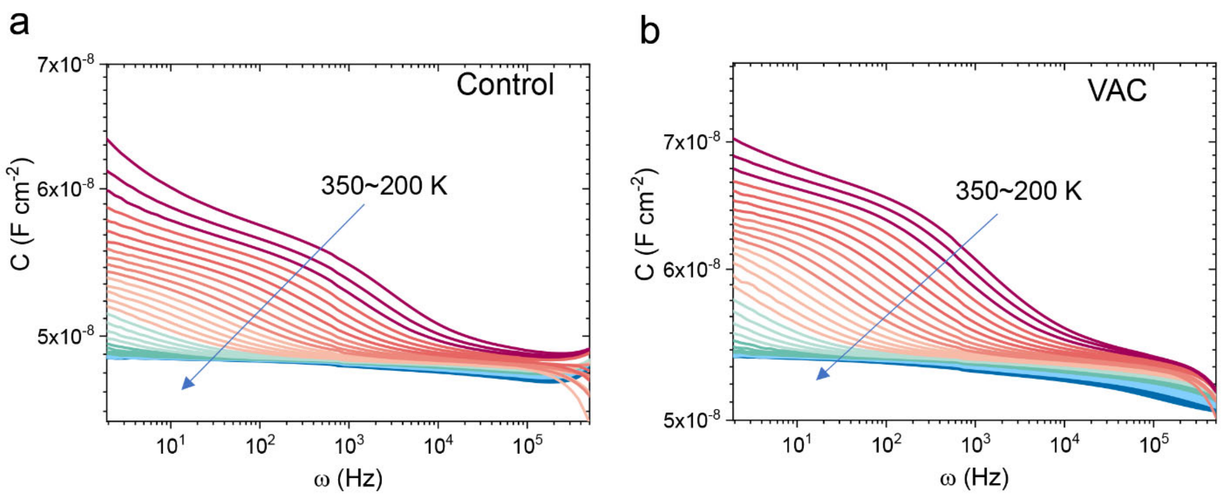

Supplementary Figure 17. Temperature dependent capacitance spectra deduced from admittance spectroscopy. a, Control device. **b**, VAC-treated device. The range of temperature is from 350 K to 200 K.

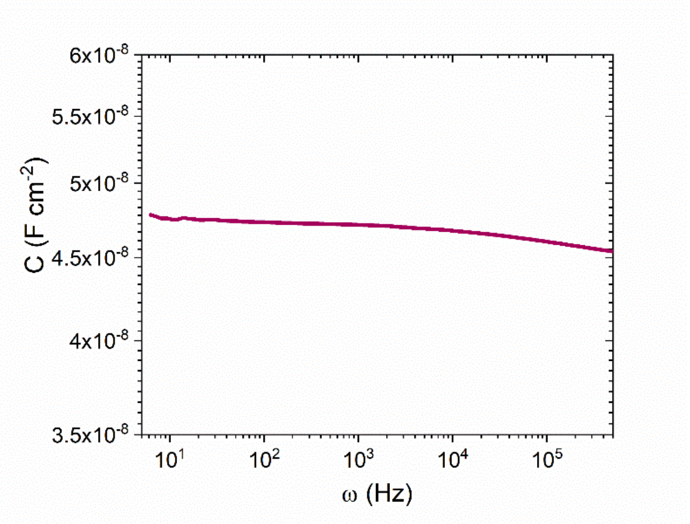

Supplementary Figure 18. Capacitance spectra (room temperature) deduced from admittance spectroscopy for the devices with only charge transport layers, *i.e.* with a configuration of ITO/NiO_x/PVK/TPBi/LiF/Al. This result indicates that the capacitance is almost independent of frequency, which is significantly different from the perovskite-based devices under the same temperature in Supplementary Figure 15.

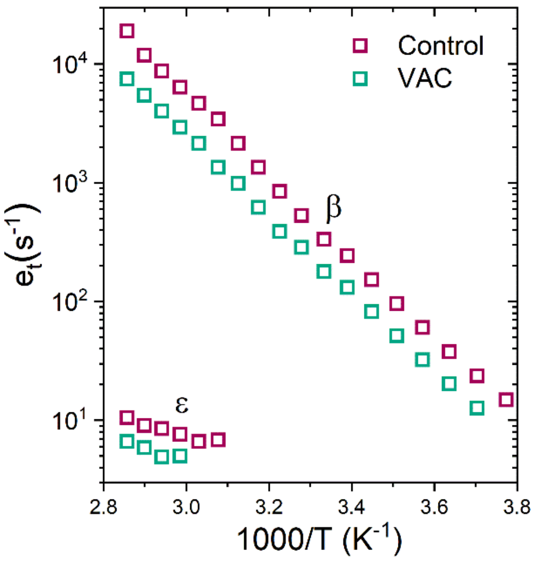

Supplementary Figure 19. Ion migration rates deduced from impedance spectroscopy. Two different mobile ions, β and ε, are visible.

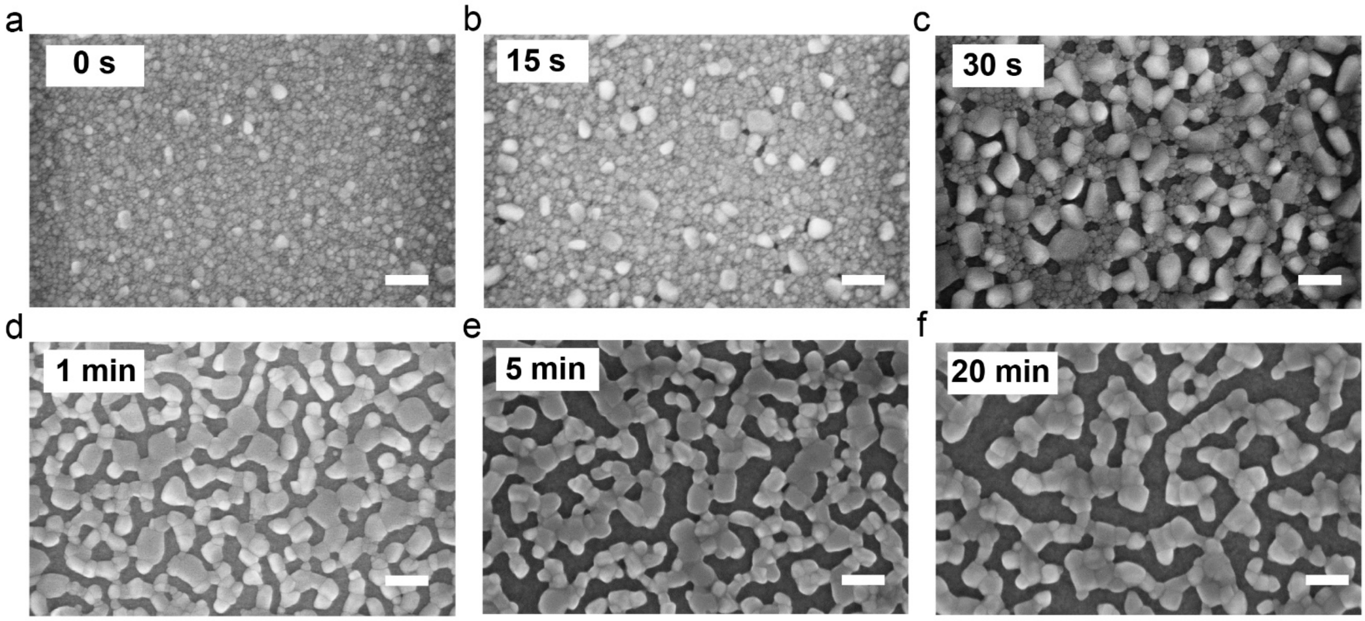

Supplementary Figure 20. Top-view SEM images showing the growth/coalescence of grains during the VAC treatment. a, pristine film (0 s). **b**, 15 s. **c**, 30 s. **d**, 1 min. **e**, 5 min. **f**, 20 min. The scale bar is 300 nm.

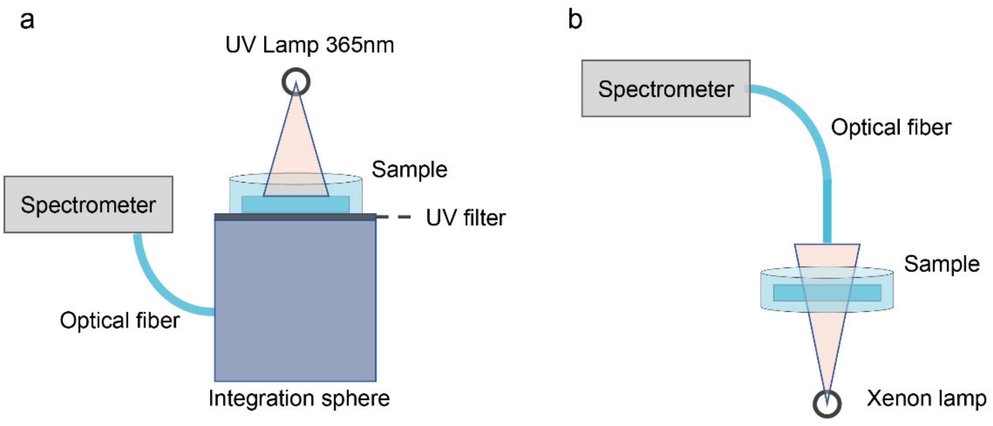

Supplementary Figure 21. Illustrations of the setups for in-situ measurements. **a**, in-situ PL. **b**, in-situ transmittance.

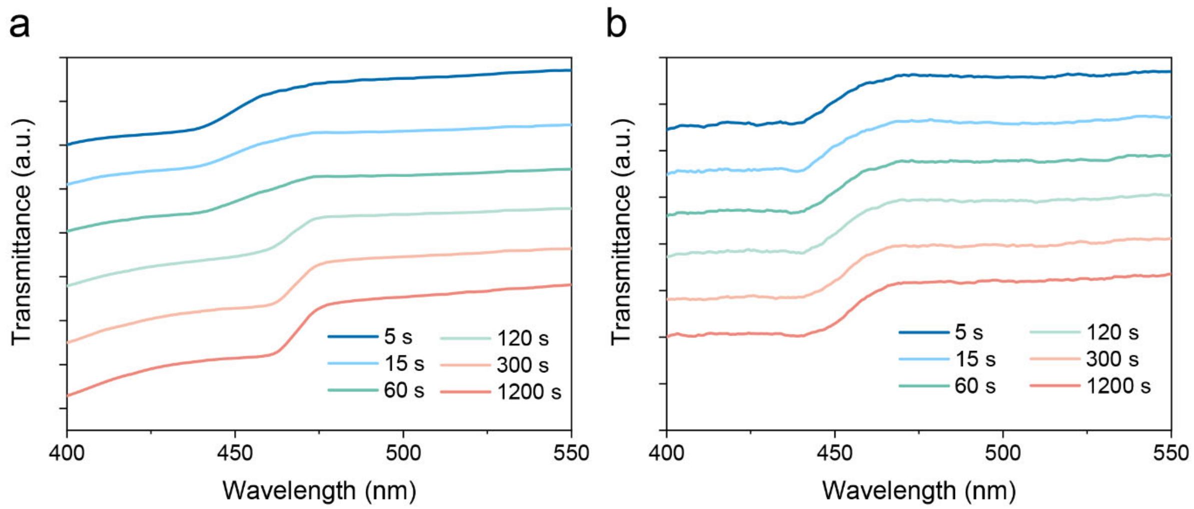

Supplementary Figure 22. In-situ transmittance spectra for monitoring the evolution of crystal growth in different atmosphere. a, With DMF vapour. **b**, Without DMF vapour.

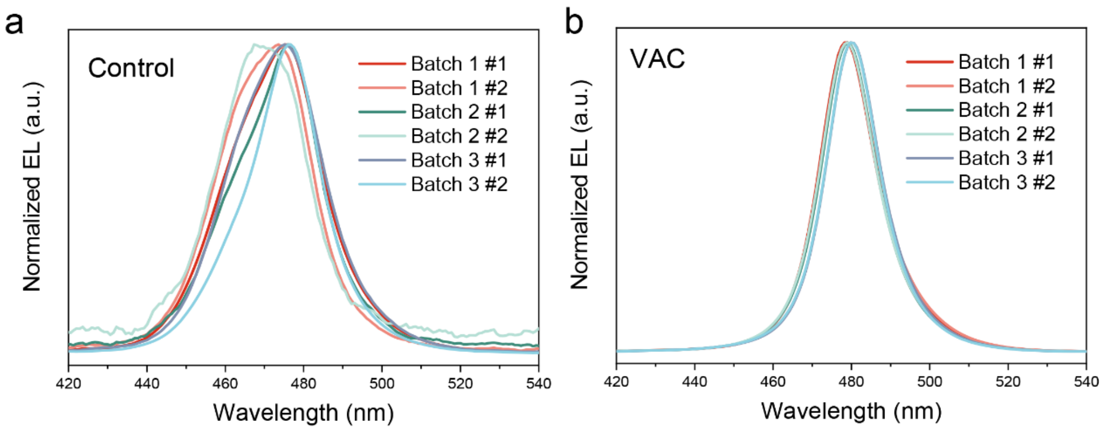

Supplementary Figure 23. EL spectra for devices prepared from different batches. a, Control devices. **b**, VAC-treated devices (20 min). The EL spectra for all the devices are extracted at 3 V.

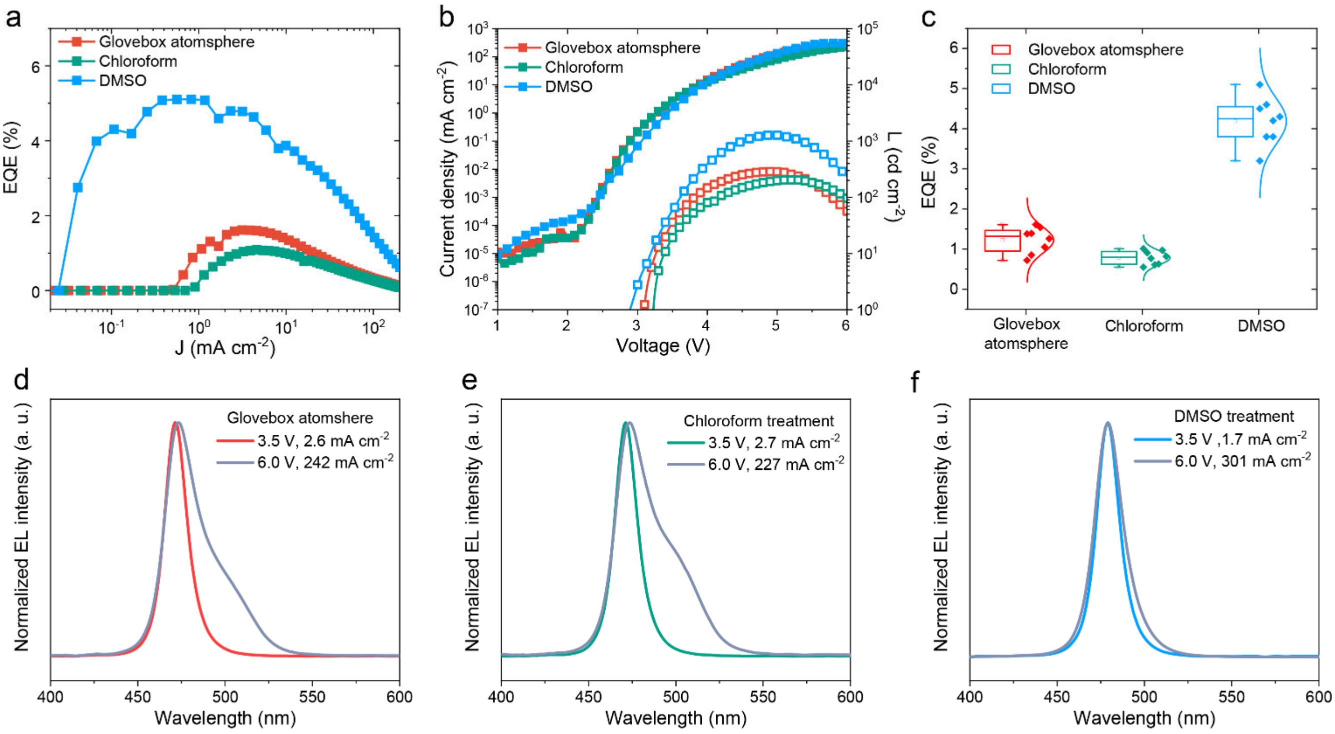

Supplementary Figure 24. Representative characteristics of VAC-treated PeLED devices with different vapour atmosphere (chloroform, DMSO or glovebox atmosphere). a, *J-EQE*. **b,** *J-V-L*. **c,** Peak EQE distribution. **d-f**, EL spectral evolution for the control devices (in the glovebox atmosphere) (**d**); the devices treated with chloroform (**e**) and the devices treated with DMSO (**f**). The EL spectra are collected at 3.5 V and 6.0 V respectively. The optimized VAC treatment duration for DMSO is around 20 min.

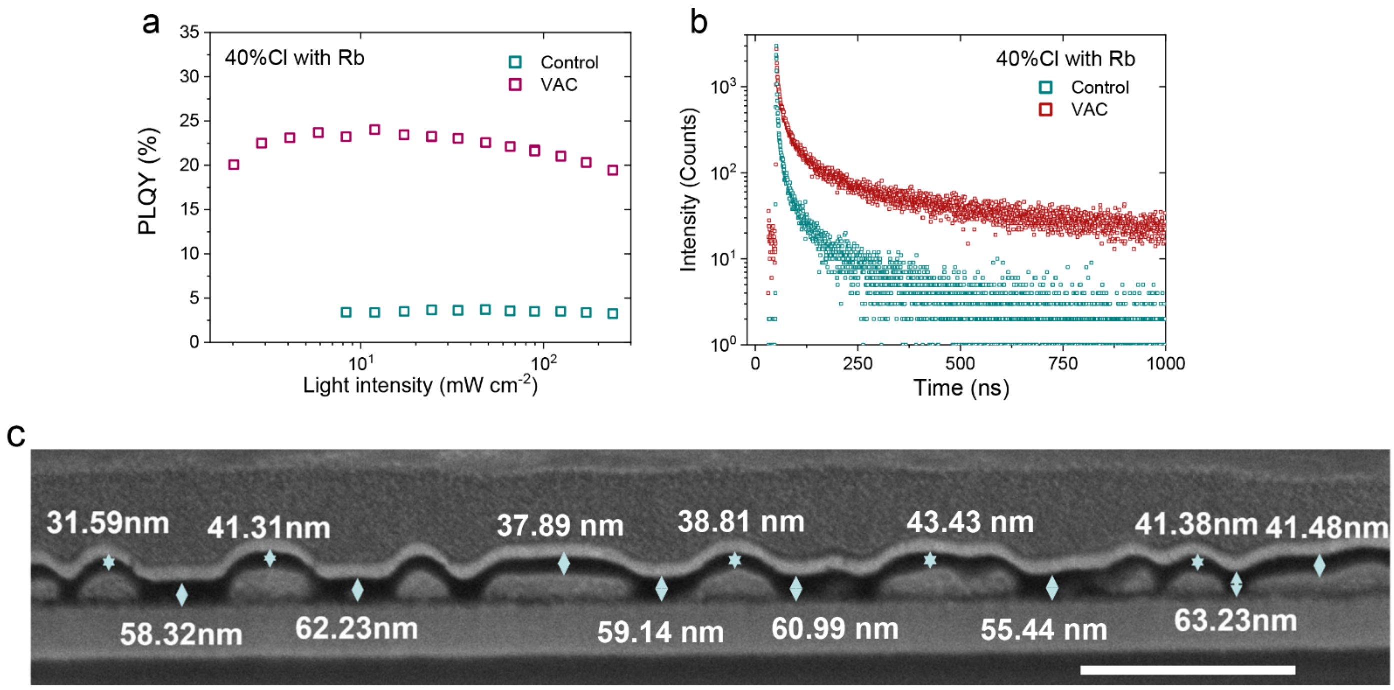

Supplementary Figure 25. Rb-passivated perovskite thin films and devices a, Fluence-dependent PLQYs for the film without (control) and with VAC treatment. **b,** PL decay measured by TCSPC. **c**, a STEM-HAADF cross-sectional image showing that the thickness of the organic layer is different on hole injection layers compared with that on perovskite grains. The scale bar is 500 nm.

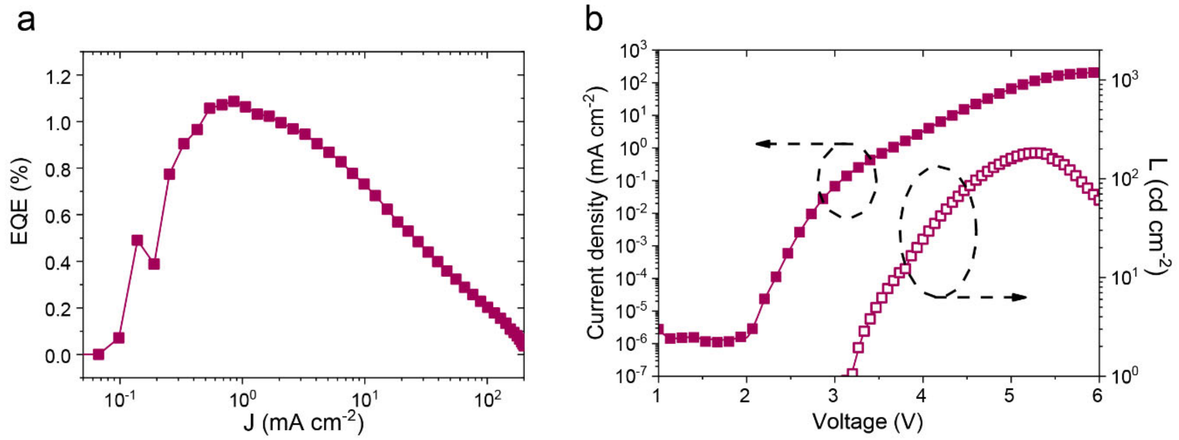

Supplementary Figure 26. Representative characteristics of PeLED devices with Rb^+ addition without VAC-treatment. a. *J-EQE*. **b.** *J-V-L*. The stoichiometry of Rb^+: Cs^+: FA^+: Pb^{2+}: $[Br_{1-x}+Cl_x]^-$: TTDDA is equal to 0.1:1.2: 0.2: 1: 3.5: 0.1 (x = 40%).

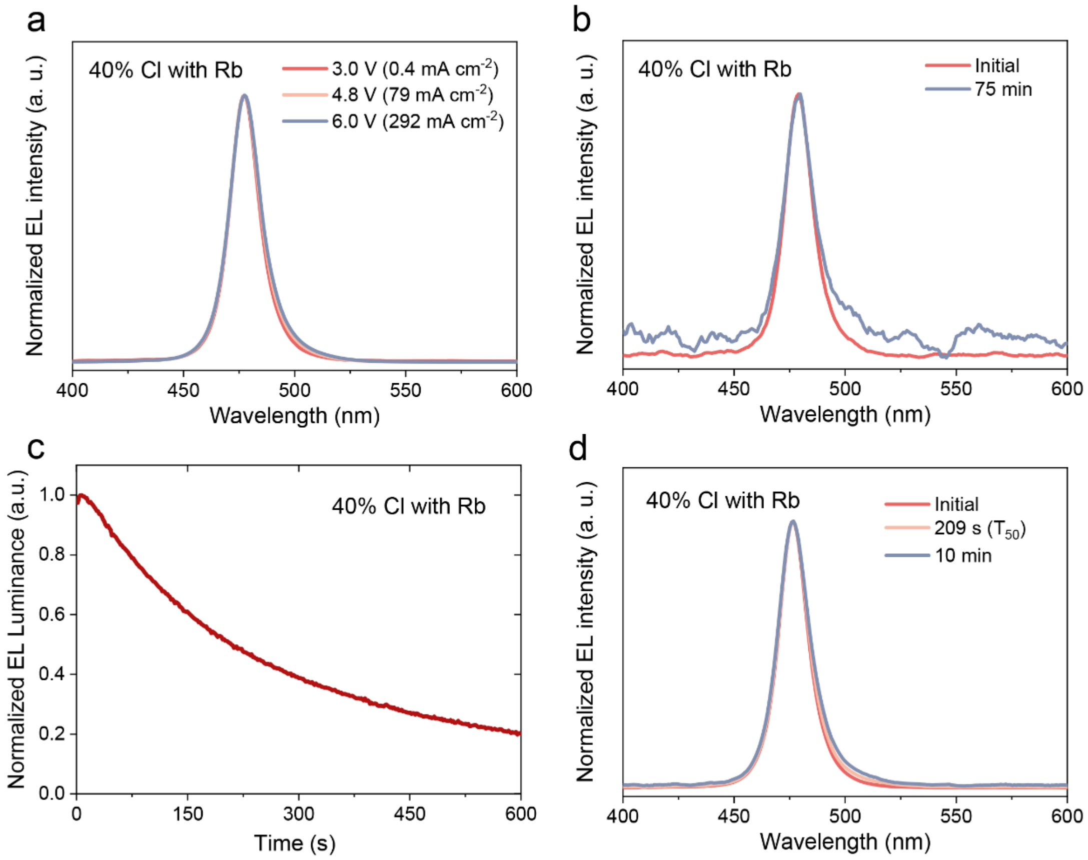

Supplementary Figure 27. Spectral and operational stability of Rb-passivated perovskite thin films (40% Cl) with VAC treatment. a, EL spectra during voltage sweep. **b**, Spectral evolution after 75 min of operation at a constant current density of 0.1 mA cm^2 (~3 V, with initial luminance of ~10 cd m^{-2}). **c**, Operational stability at a constant current density of ~1.34 mA cm^{-2}. The initial luminescence value is ~102 cd m^{-2}. **d**, The initial EL spectrum and those collected at T_{50} and after 10 minutes' operation.

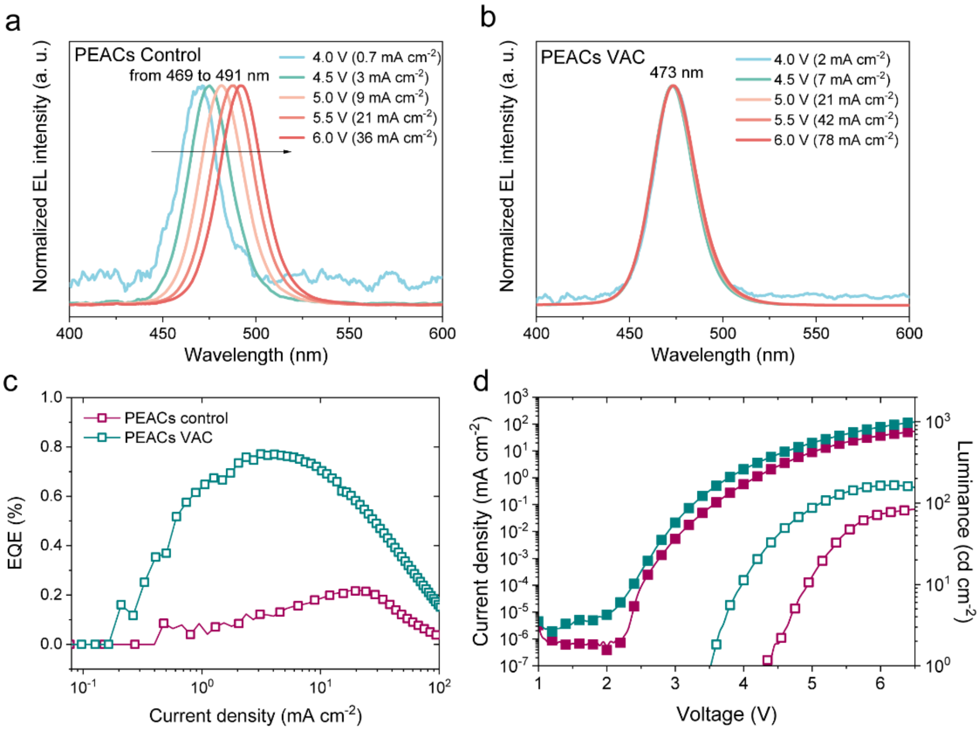

Supplementary Figure 28. Device performance and spectral evolution of PEA-CsPbBr$_{(0.7}$Cl$_{0.3)3}$ with and without VAC treatment. a, Normalized EL spectra (4~6 V) of PEA-incorporated control device showing its poor stability. **b**, Normalized EL spectra (4~6 V) of PEA-incorporated device after VAC-treatment showing a stabilized spectrum under electrical bias. **c**, EQE as a function of current density. **d**, Current density and luminance as a function of voltage. The duration for VAC-treatment in this case is 5 min.

Supplementary Table 1. Summary of state-of-the-art sky-blue PeLEDs (CIE_y>0.15) with good spectral stability.

	Peak Wavelength (nm)	CIE (x, y)	FWHM (nm)	Peak EQE (%)	Maximum Luminance (cd m^{-2})	Ref.
colloidal nano-crystals	488	NA	23	1.41	830	*Adv. Mater.* **30**, 1706226 (2018).
	485	(0.085, 0.245)	23	2.62	1200	*Adv. Funct. Mater.* **30**, 1908339 (2020)
low-dimensional perovskite bulk films	485	(0.16, 0.23)	NA	2.6	200	*ACS Appl. Mater. Interfaces* **9**, 29901 (2017)
	490	NA	28	1.5	2480	*Nat. Commun.* **9**, 3541 (2018).
	487	NA	27	6.2	3340	*Chem. Mater.* **31**, 83 (2019).
	485	(0.08, 0.21)	23	11.0	9040	*Nat. Commun.* **10**, 5633 (2019)
	483	(0.094,0.184)	26	9.5	770	*Nat. Photon.* **13**, 76 (2019)

Supplementary Table 2. Summary of state-of-the-art blue PeLEDs (CIE_y<0.15) with good spectral stability.

	Peak Wavelength (nm)	CIE (x, y)	FWHM (nm)	Peak EQE (%)	Maximum Luminance (cd m^{-2})	Ref.
colloidal nano-crystals	470	(0.121, 0.077)	17.1	1.46	389	*Joule* **2**, 2421 (2018).
	466	NA	17.9	2.12	245	
	471	(0.129, 0.087)	17	6.3	465	*ACS Energy Lett.* **5**, 793 (2020)
	463	NA	17	3.3	569	*Sci. Bull.* 55, 1150 (2020)
	479	(NA, 0.13)	20	12.3	~400	*Nat. Nanotechnol.* https://doi.org/10.1038/s41565-020-0714-5 (2020).
low-dimensional perovskite bulk films	465	(0.14, 0.05)	25	2.6	211	*Adv. Mater.* **31**, 1904319 (2019).
	475	(0.115, 0.099)	20	1.35	100.3	*Nat. Commun.* **10**, 1868 (2019).
	474	(0.11, 0.10)	27.7	4.0	150	*Nat. Photon.* **13**, 76 (2019)
	478	(0.12, 0.14)	NA	6.3	<200	*Nat. Commun.* **11**, 3694 (2020)
3D perovskites	477	(0.107, 0.115)	18	11.0	2180	This work
	467	(0.130, 0.059)	18	5.5	330	
		(0.14, 0.08)				NTSC blue
		(0.131, 0.046)				Rec. 2020 blue

Supplementary Table 3. Summary of deduced signature including ion-migration activation energy (E_A), ion-diffusion coefficient at 300K (D_{300K}), and mobile ion concentration (N_i) from impedance spectra.

	E_A (eV)	D_{300K} (cm^2 s^{-1})	N_i (cm^{-3})
ε (control)	0.21	8.3×10^{-13}	5.4×10^{16}
ε (VAC)	0.24	7.8×10^{-13}	1.9×10^{16}
β (control)	0.70	9.1×10^{-11}	1.8×10^{16}
β (VAC)	0.68	7.5×10^{-11}	1.6×10^{16}

Reference

1. Anthopoulos, T. D. *et al.* Highly efficient single-layer dendrimer light-emitting diodes with balanced charge transport. *Appl. Phys. Lett.* **82**, 4824-4826 (2003).

2. Dai, X. *et al.* Solution-processed, high-performance light-emitting diodes based on quantum dots. *Nature* **515**, 96-99 (2014).

3. Wang, J. *et al.* High efficiency green phosphorescent organic light-emitting diodes with a low roll-off at high brightness. *Org. Electron.* **14**, 2854-2858 (2013).

4. Cao, J. *et al.* Low-temperature solution-processed NiO_x films for air-stable perovskite solar cells. *J. Mater. Chem. A*, **5**, 11071-11077 (2017).

5. Cao, Y. *et al.* Perovskite light-emitting diodes based on spontaneously formed submicrometre-scale structures. *Nature* **562**, 249-253 (2018).

6. Xu, W. *et al.* Rational molecular passivation for high-performance perovskite light-emitting diodes. *Nat. Photon.* **13**, 418-424 (2019).
